# BCG HSP70 Reprograms Macrophages via Central Trained Immunity to Suppress Prostate Cancer

**DOI:** 10.1002/advs.76886

**Published:** 2026-08-05

**Authors:** Peng Liu, Kangkang Liu, Keruo Wang, Heyang Guan, Jiaming Fan, Taipeng Li, Jiaru Hao, Zeyuan Wang, Yongchao Yan, Jiatong Chen, Yuan Ma, Yang Yang, Yuanjie Niu

**Affiliations:** ^1^ Nankai University Tianjin China; ^2^ Department of Urology, Tianjin Institute of Urology The Second Hospital of Tianjin Medical University Tianjin China; ^3^ Tianjin Medical University General Hospital Tianjin China; ^4^ Haihe Laboratory of Sustainable Chemical Transformations Tianjin China; ^5^ School of Chemical Engineering and Technology Hebei University of Technology Tianjin China

**Keywords:** BCG HSP70, macrophage, trained immunity, tumor environment

## Abstract

Trained immunity offers a promising yet clinically challenging strategy for cancer immunotherapy, as current inducers such as BCG pose safety concerns and their active components remain ill‐defined. BCG‐derived heat shock protein 70 (BCG HSP70, Dnak) has previously been recognized only as an immune adjuvant, while its role in trained immunity remains unexplored. This study identifies Dnak as a safe, defined inducer that establishes durable central trained immunity through epigenetic and metabolic reprogramming of bone marrow hematopoietic stem cells, conferring protection against bacterial infection. In prostate cancer models, a single Dnak pre‐treatment significantly inhibits tumor growth—an effect transferable via bone marrow transplantation and dependent on tumor‐associated macrophages. Mechanistically, Dnak‐trained macrophages exhibit enhanced glycolysis, mTOR/HIF‐1α pathway activation, and M1‐like polarization. Notably, O‐GlcNAcylation emerges as a previously unrecognized regulator of trained immunity. Furthermore, combining Dnak‐induced trained immunity with a tumor vaccine achieves superior tumor control, reshapes the tumor microenvironment, and generates durable memory T cell responses upon rechallenge. These findings establish BCG HSP70 as a safe and effective protein‐based trained immunity inducer with translational potential for cancer immunotherapy.

## Graphical abstract

BCG‐derived HSP70 (Dnak) safely induces central trained immunity via epigenetic, metabolic, and O‐GlcNAcylation reprogramming of bone marrow progenitors, driving tumor‐associated macrophages toward an M1‐like phenotype to suppress prostate cancer.

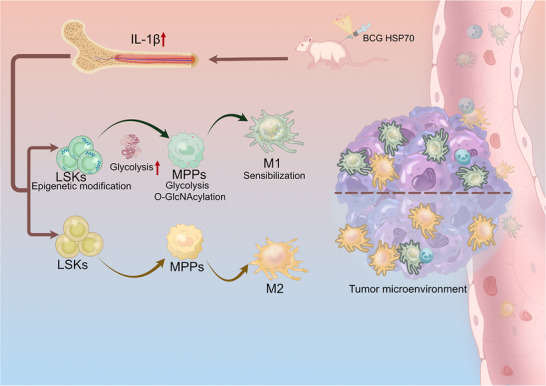

## 1 Introduction

Beyond the classical framework of adaptive immune memory, the concept of trained immunity posits that the innate immune system can also develop a form of functional long‐term memory [1, 2, 3]. This process is characterized by persistent epigenetic, metabolic, and functional reprogramming of innate immune cells following an initial stimulus, enabling a heightened and more rapid response upon secondary homologous or heterologous challenge [4, 5, 6]. Notably, trained immunity can be established not only in mature circulating myeloid cells but also at the level of bone marrow (BM) myeloid progenitors and even hematopoietic stem and progenitor cells (HSPCs), leading to a systemic and durable modulation of immunity [7, 8]. This presents a novel avenue for cancer immunotherapy. In solid tumors such as prostate cancer, the immunosuppressive microenvironment poses a major therapeutic hurdle, with myeloid‐derived innate immune cells playing a pivotal role in promoting tumor growth, angiogenesis, and immune evasion [9, 10, 11]. However, reversing this immune‐tolerant state remains a clinical challenge, as malignant cells can manipulate immune tolerance at the BM level and direct intratumoral drug delivery is difficult [12, 13, 14]. Therefore, a systemic intervention capable of reprogramming the developmental trajectory of myeloid cells at the bone marrow (BM) level, priming them to adopt anti‐tumor phenotypes upon tumor infiltration, would hold significant therapeutic value [15, 16, 17, 18]. Trained immunity represents a potential mechanism to achieve this goal [19]. However, its application in tumor immunotherapy remains at an early stage, and safe, well‑controlled inducers are still lacking.

Bacillus Calmette‐Guérin (BCG) is the most extensively studied and clinically applied inducer of trained immunity [20, 21, 22, 23, 24]. It has been reported that intravenous, but not subcutaneous, administration of BCG can elicit systemic anti‐tuberculosis trained immunity [21]. However, systemic administration of live BCG carries significant safety risks, including severe or even life‐threatening adverse effects such as persistent high fever, systemic inflammatory response syndrome, or sepsis [25]. Furthermore, while studies indicate that inactivated BCG retains the ability to induce trained immunity, suggesting this capacity is mediated by specific components rather than live bacteria, BCG, as a whole pathogen, is highly complex [21]. To date, only the muramyl dipeptide (MDP) component has been identified, leaving it unclear whether other critical molecules are involved [26]. This dependence on systemic administration, coupled with an incomplete understanding of its active components, collectively limits the translational application of BCG in broader solid tumor immunotherapy.

Notably, BCG‐derived heat shock protein 70 (HSP70), known as Dnak, a highly conserved protein, exhibits unique immunomodulatory potential [27]. Previous studies have confirmed that BCG HSP70 itself is a safe and potent immunoadjuvant, with a significantly superior safety profile compared to whole BCG. Mechanistically, it engages multiple innate immune receptors, including TLR4, CD40, and CD91, on antigen‐presenting cells, promoting dendritic cell maturation and facilitating antigen cross‐presentation. These interactions converge on downstream signaling pathways such as NF‐κB [27], which constitute key signaling networks that initiate trained immunity [28].

Given the safety concerns associated with whole BCG and the unexplored potential of its individual components, we sought to determine whether BCG‐derived HSP70 (Dnak) could serve as a defined and safe inducer of trained immunity. Our findings demonstrate that Dnak safely induces durable central trained immunity, reprograms tumor‐associated macrophage (TAM) function, and suppresses prostate cancer through a previously unappreciated mechanism involving O‐GlcNAcylation. Together, these results establish Dnak as a candidate for next‐generation trained immunity‐based cancer immunotherapy.

## 2 Results

### 2.1 Dnak Shows a Superior Biosafety Profile Compared to Other Trained Immunity Inducers

To evaluate biosafety, mice were administered either PBS (vehicle control), Dnak, MDP, β‐glucan, or BCG at varying doses. Body temperature, inflammatory cytokine levels, liver function, and kidney function were monitored (Figures S1–S3).

In the low‑dose regimen (0.2 mg per mouse for Dnak, MDP, and β‑glucan; 2 × 10^5^ CFU for BCG) (Figure S1A), none of the treated groups displayed any abnormal elevation in core body temperature (Figure S1B). Histological examination of liver and kidney sections (H&E staining) revealed no obvious pathological changes (Figure S1C). Serum biochemical parameters, including hepatic transaminases (ALT, AST) and renal function markers (BUN, creatinine), remained within normal ranges at both 24 h (Figure S1D) and day 7 (Figure S1E) post‑injection, indicating no detectable organ injury at this dose level.

At the intermediate dose (1 mg per mouse for the purified ligands; 1 × 10^6^ CFU for BCG), distinct responses emerged within 24 h post‑injection (Figure S2A). MDP‑treated mice showed a significant elevation in body temperature, accompanied by markedly increased serum levels of tumor necrosis factor‑α (TNF‑α) and monocyte chemoattractant protein‑1 (MCP‑1) (Figure S2B,C). H&E staining revealed that β‑glucan and BCG, but not Dnak or MDP, induced hepatic congestion, inflammatory cell infiltration, and pale hepatocytes at 24 h (Figure S2D). Furthermore, serum biochemical assays at both 24 h (Figure S2E) and day 7 (Figure S2F) demonstrated significant elevations in ALT or AST levels in the β‑glucan and BCG groups, indicating persistent hepatic dysfunction.

At the highest dose (2 mg per mouse for Dnak, MDP, and β‑glucan; 2 × 10^6^ CFU for BCG), the findings were consistent with those observed at the intermediate dose (Figure S3A,B). Again, β‑glucan and BCG, but not Dnak or MDP, caused significant hepatic impairment, as evidenced by elevated ALT or AST levels at both 24 h and day 7, with no concurrent renal dysfunction (Figure S3C–E).

In summary, while Dnak and MDP did not induce hepatorenal toxicity at any tested dose, MDP triggered a significant acute febrile and inflammatory response at the intermediate and highest doses. In contrast, β‐glucan and BCG, particularly at intermediate and high doses, exhibited selective and persistent hepatotoxicity without affecting renal function. These findings highlight distinct biosafety profiles among the tested immunomodulators, with Dnak showing the most favorable safety margin in this model.

### 2.2 Dnak Induces Trained Immunity via Epigenetic Modifications in LSK Cells

**FIGURE 1:**
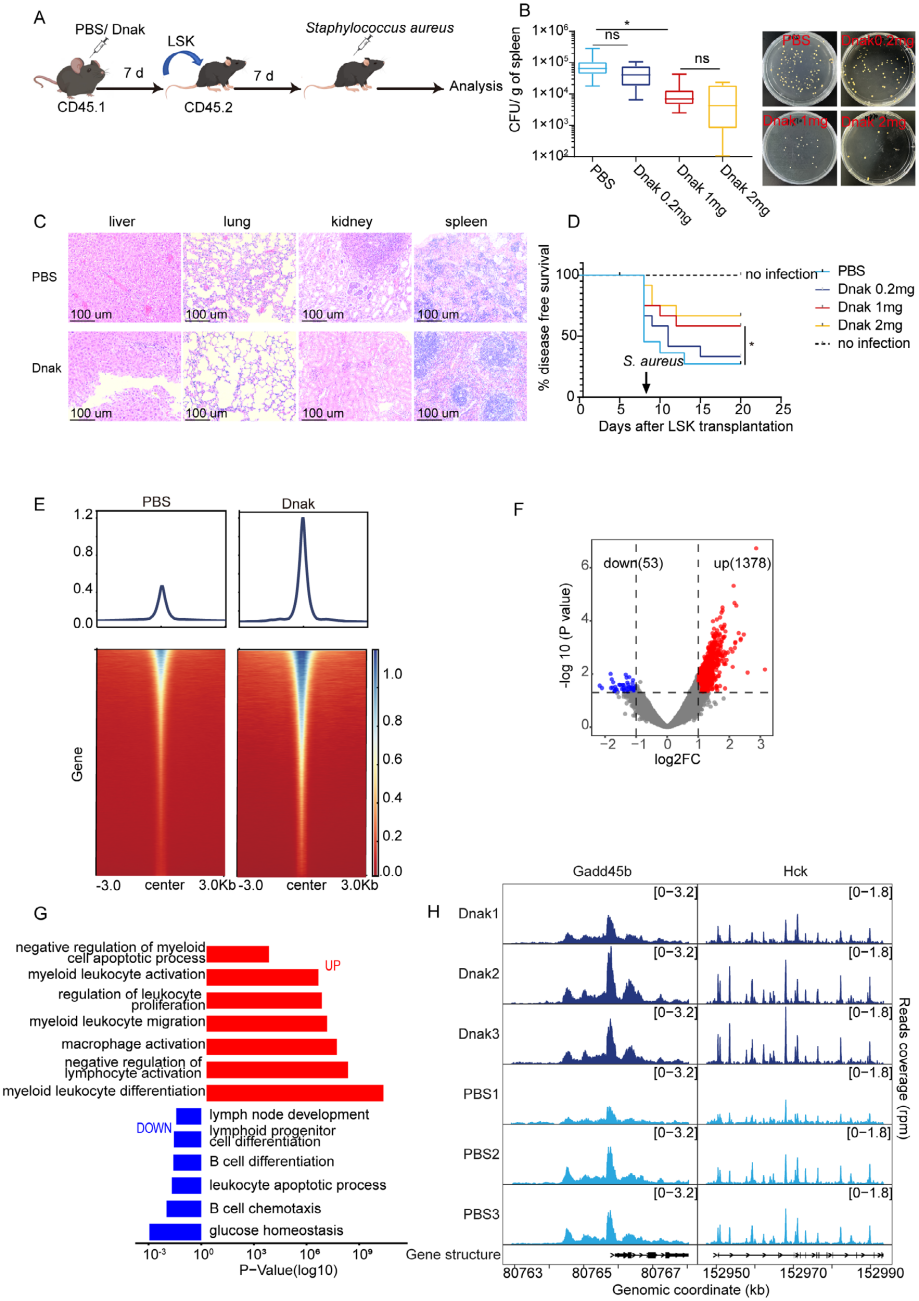
Dnak administration induces epigenetic modifications in LSK cells, enhancing anti‐infective responses. (A) Schematic of the experimental design. CD45.1 mice were injected intraperitoneally (i.p.) with PBS or Dnak at 0.2, 1, or 2 mg per mouse. One week later, LSK cells from these donors were sorted and transplanted into lethally irradiated CD45.2 recipient mice. Seven days after transplantation, recipient mice were infected i.p. with 1 × 10^7^ colony‐forming units (CFUs) of Staphylococcus aureus. (B) Bacterial burden in the spleen 16 h post‐infection for mice described in (A) (n = 8). (C) Representative H&E‑stained sections of liver, lung, kidney, and spleen collected 16 h post‑infection from mice treated with PBS or Dnak (1 mg, i.p.) as described in (A). Scale bar: 100 µm. (D) Disease‐free survival curves of mice described in (A) (n = 12). (E–H) WT mice received a single intraperitoneal injection of Dnak (1 mg) or PBS. Seven days later, bone marrow cells were harvested, and LSK cells (Lin^−^Sca‑1^+^c‑Kit^+^) were FACS‑sorted (gating strategy as described in Figure S4). Purified LSK cells were subjected to ATAC‑seq to assess genome‑wide chromatin accessibility (n = 3). (E) Dnak treatment enhances chromatin accessibility at transcription start sites (TSS). Average read density distribution of ATAC‐seq signals centered on TSS regions (±3.0 kb) in LSK cells from PBS‐treated (left) and Dnak‐treated (right) mice. Lower panels: heatmaps of ATAC‐seq signals aligned to TSSs, spanning ±3.0 kb. Color intensity represents chromatin accessibility levels. (F) Scatterplot analysis of ATAC‐seq peaks in LSK cells from Dnak‐treated versus PBS‐treated mice. Significantly increased open chromatin regions (OCRs) are highlighted in red, and decreased OCRs in blue. Numbers of differentially accessible peaks are indicated. Data are plotted on a log2 scale. (G) Selected Gene Ontology (GO) biological process terms associated with genes near gained (red) and lost (blue) OCRs. The complete list is provided in Table S1. (H) Representative IGV tracks showing ATAC‐seq peaks of gained OCRs in LSK cells from Dnak (1 mg, i.p.) treated mice compared to PBS controls. Data are presented as mean ± SD. For comparisons between two groups, an unpaired two‑tailed Student's t‑test was applied when data met normal distribution assumptions; otherwise, the Mann–Whitney U test was used. Multiple‐group comparisons were analyzed by one‐way ANOVA with Tukey's post hoc test or by Kruskal–Wallis test with Dunn's multiple‐comparison test. Survival curves were compared using the log‐rank test. ^*^ *p* < 0.05; ^**^ *p* < 0.01; ^***^ *p* < 0.001; ns, not significant.

To investigate whether Dnak can induce trained immunity in vivo, we employed a bacterial infection model in immunocompromised mice during hematopoietic stem cell (HSC) transplantation [29]. Seven days after a single intraperitoneal (i.p.) injection of Dnak at varying doses (0.2, 1, or 2 mg) into CD45.1 mice, we harvested and transplanted their Lineage‐negative, c‐Kit^+^, Sca‐1^+^ (LSK) cells into lethally irradiated CD45.2 recipients. Seven days post‐transplantation, when the mice remained immunocompromised, they were challenged with 1 × 10^7^ colony‐forming units (CFUs) of the *Staphylococcus aureus* (Figure 1A).

Mice receiving 0.2 mg Dnak exhibited only a modest, non‑significant reduction in bacterial load compared with PBS‑treated controls. In contrast, mice receiving 1 or 2 mg of Dnak showed significantly lower splenic bacterial counts, with the 2 mg dose achieving the greatest reduction, indicating a dose‑response relationship (Figure 1B). Histopathological examination of major organs revealed that 1 mg Dnak treatment mitigated infection‑induced tissue damage (Figure 1C). To evaluate the protective effect of Dnak, we monitored post‑challenge survival of transplanted mice for 25 days. Dnak pretreatment improved survival in a dose‑dependent manner. The 0.2 mg group showed no significant benefit, whereas the 1 mg and 2 mg groups exhibited significantly higher survival rates (Figure 1D).

Together, these data indicated that pre‐stimulation with Dnak induces persistent functional changes in LSK cells, conferring a significantly improved response against secondary bacterial infection.

To determine whether the enhanced anti‐infective capacity conferred by Dnak pre‐immunization is attributable to epigenetic modifications in LSK cells, we analyzed changes in chromatin accessibility in Dnak‐exposed LSK cells using assay for transposase‐accessible chromatin with high throughput sequencing (ATAC‐seq). The heatmap panel illustrated the detailed distribution of chromatin accessibility across ±3.0 kb upstream and downstream of transcription start sites (TSS), confirming that Dnak stimulation globally enhanced chromatin opening at TSS regions, as reflected by the more concentrated and intense accessibility signals relative to the control (Figure 1E). This analysis revealed 1,378 increased and 53 decreased open chromatin regions (OCRs) one week after Dnak exposure (Figure 1F). Gene Ontology (GO) analysis of genes associated with newly induced OCRs enriched for terms related to innate immune response and myeloid differentiation (Figure 1G; Table S1). Examples of genes within these pathways were shown in Figure 1H. Conversely, GO analysis of genes with decreased OCRs revealed terms associated with adaptive immune response (Figure 1G; Table S1). Motif analysis of the differential peaks identified transcription factors in public databases that matched these motifs. Many of these factors were associated with innate immunity and myeloid differentiation, suggesting that the altered chromatin accessibility regulates trained immunity (Table S2). Collectively, these data demonstrate that Dnak induces trained immunity by inducing epigenetic modifications in LSK cells.

### 2.3 Dnak Enhances Myelopoiesis and Alters the Transcriptome of Multipotent Progenitors (MPPs)

**FIGURE 2:**
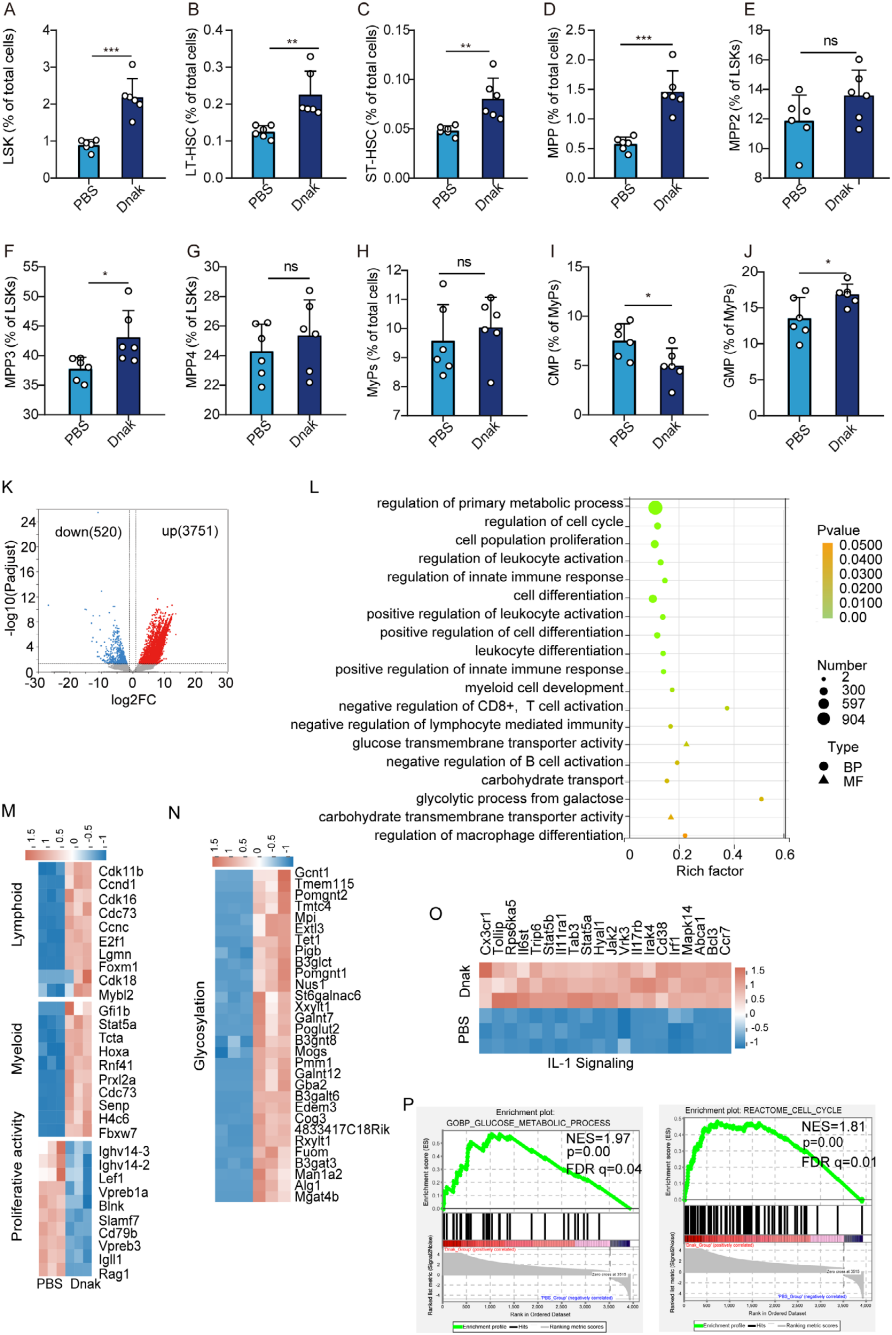
Dnak administration induces myelopoiesis and alters the transcriptome of multipotent progenitors (MPPs). (A–J) WT mice received a single intraperitoneal (i.p.) injection of Dnak 1 mg or PBS. Bone marrow (BM) analysis was performed 7 days post‐administration. The detailed gating strategy is shown in Figure S4. (A–D) Frequency of LSK cells (Lin^−^ c‐Kit^+^ Sca‐1^+^) (A), long‐term hematopoietic stem cells (LT‐HSCs; CD48^−^ CD150^+^ LSK) (B), short‐term hematopoietic stem cells (ST‐HSCs; CD48^−^ CD150^−^ LSK) (C), and multipotent progenitors (MPPs; CD48^+^ CD150^−^ LSK) (D) within total BM cells (n = 6). (E–G) Frequency of MPP2 (Flt3^−^ CD150^+^ CD48^+^LSK) (E), MPP3 (Flt3^−^ CD150^−^ CD48^+^LSK) (F), and MPP4 (Flt3^+^ CD150^−^ CD48^+^LSK) (G) subsets within the LSK population (n = 6). (H) Frequency of myeloid progenitors (MyPs; Lin^−^ c‐Kit^+^ Sca‐1^−^) within total BM cells (n = 6). (I–J) Frequency of common myeloid progenitors (CMPs; Lin^−^ c‐Kit^+^ Sca‐1^−^ CD16/32^−^ CD34^+^) (I) and granulocyte‐macrophage progenitors (GMPs; Lin^−^ c‐Kit^+^ Sca‐1^−^ CD16/32^+^ CD34^+^) (J) within the MyP population (n = 6). (K–P) Transcriptome analysis of MPPs sorted from BM of mice 7 days after Dnak (1 mg, i.p.) or PBS administration (n = 3). (K) Volcano plot showing differential gene expression in MPPs from Dnak‐treated mice compared to PBS controls. Significantly upregulated (red) and downregulated (blue) genes are highlighted (false discovery rate [FDR] < 0.05). Dashed lines indicate significance thresholds. (L) Selected Gene Ontology (GO) biological process terms enriched among upregulated genes. The complete list is provided in Table S3. (M) Heatmap showing expression of genes related to cell proliferation, myeloid lineage, and lymphoid lineage. (N) Heatmap showing expression of genes involved in glycosylation. (O) Heatmap showing expression of genes involved in IL‐1 signaling. (P) Gene set enrichment analysis (GSEA) plots for glucose metabolic process and cell cycle‐related gene sets. NES, normalized enrichment score. FDR q‐values are indicated. Data are presented as mean ± SD. For comparisons between two groups, an unpaired two‑tailed Student's t‑test was applied when data met normal distribution assumptions; otherwise, the Mann–Whitney U test was used. Multiple‐group comparisons were analyzed by one‐way ANOVA with Tukey's post hoc test or by Kruskal–Wallis test with Dunn's multiple‐comparison test. ^*^ *p* < 0.05; ^**^ *p* < 0.01; ^***^ *p* < 0.001; ns, not significant.

To explore the impact of Dnak on LSK cells, we performed flow cytometric analysis on BM cells from mice following i.p. injection of Dnak or PBS. One day post‐immunization, we observed an increase in long‐term hematopoietic stem cells (LT‐HSCs; CD48^−^ CD150^+^ LSK), as well as a significant increase in the proportion of myeloid‐biased multipotent progenitors 2 (MPP2; Flt3^−^ CD48^+^ CD150^+^ LSK) and myeloid progenitors (MyPs; Lin^−^ c‐Kit^+^ Sca‐1^−^) (Figure S4), suggesting a transient boost in myelopoiesis. Seven days after Dnak administration, we found increased proportions of total hematopoietic progenitors (LSKs; Lin^−^ c‐Kit^+^ Sca‐1^+^), LT‐HSCs, short‐term hematopoietic stem cells (ST‐HSCs; CD48^−^ CD150^−^ LSK), and multipotent progenitors (MPPs; CD48^+^ CD150^−^ LSK) (Figure 2A–D). Analysis of MPP subsets revealed a predominant expansion of the myeloid‐biased MPP3 (Flt3^−^ CD48^+^ CD150^−^ LSK) subset (Figure 2E–G). Analysis of myeloid progenitors (MyPs) at the 7‐day time point revealed that Dnak treatment resulted in an increased proportion of granulocyte‐macrophage progenitors (GMPs; Lin^−^ c‐Kit^+^ Sca‐1^−^ CD16/32^+^ CD34^+^) within the MyP population, accompanied by a corresponding reduction in common myeloid progenitors (CMPs; Lin^−^ c‐Kit^+^ Sca‐1^−^ CD16/32^−^ CD34^+^), compared to control mice (Figure 2H–J).

To understand the impact of Dnak on the LSK population, we next performed bulk RNA sequencing (RNA‐seq) on MPPs (CD48^+^ CD150^−^ LSK) isolated from the BM of Dnak‐ or PBS‐treated (i.p.) wild‐type mice. We found significant differences in gene expression 7 days after Dnak stimulation (3751 increased and 520 decreased genes; false discovery rate [FDR] < 0.05) (Figure 2K). GO analysis revealed overrepresentation of pathways involved in cell proliferation and myelopoiesis (Figure 2L). In contrast, pathways related to lymphocyte development and function were underrepresented (Figure 2L). Moreover, GO analysis demonstrated that pathways involved in protein modification and glycosylation were enriched in MPPs from Dnak‐treated mice (Table S3). Upregulation of myeloid lineage markers, including *Stat5a, Tcta*, and *Cdc73*, and downregulation of lymphoid lineage markers, such as *Rag1, Ighv14‐3*, and *Vpreb3*, were also observed in MPPs from Dnak‐treated mice (Figure 2M). In addition, we found increased expression of several genes associated with glycosylation and IL‐1 signaling (Figure 2N,O). Gene set enrichment analysis (GSEA) using the Molecular Signatures Database (MSigDB) hallmark gene set collection showed a significant positive correlation with cell cycle and glucose metabolic process gene sets in MPPs from Dnak‐treated mice (Figure 2P; Table S4). Collectively, these transcriptional changes indicate that Dnak induces a myeloid‐skewed transcriptional reprogramming in MPPs, characterized by enhanced expression of genes related to myelopoiesis, cell proliferation, glycosylation, and IL‐1 signaling, while suppressing lymphoid lineage genes, thereby establishing a molecular foundation for the subsequent functional and metabolic alterations associated with trained immunity.

### 2.4 Glycolysis and Glycosylation are Involved in Trained Immunity

**FIGURE 3:**
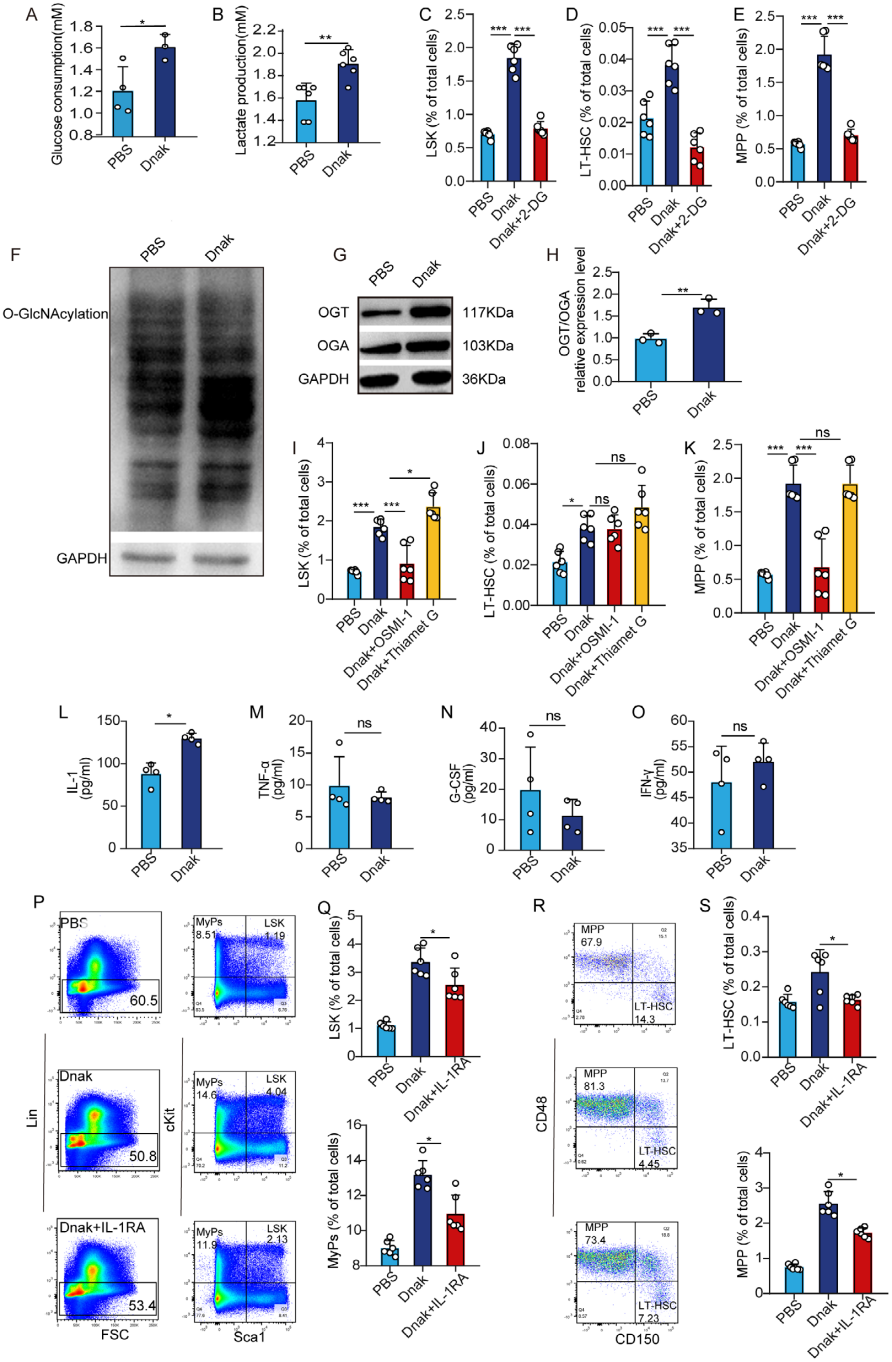
Glycolysis, O‐GlcNAcylation, and IL‐1β/TLR4 signaling mediate Dnak‐induced trained immunity. (A,B) Mice were intraperitoneally injected with PBS (vehicle) or Dnak at 1 mg per mouse. After 7 days post‑injection, bone marrow cells were harvested, and MPPs were isolated via FACS. The sorted MPPs were cultured in vitro for 24 h in complete medium, after which supernatants were collected to measure glucose consumption (A) and lactate accumulation (B) (n = 6). (C–E) Mice were treated with PBS, Dnak (1 mg, i.p.), or 2‐DG plus Dnak (1 mg, i.p.). Bone marrow single‑cell suspensions were prepared 7 days post‑injection and analyzed by flow cytometry (n = 6). (C) Frequency of LSK cells (Lin^−^ c‐Kit^+^ Sca‐1^+^) within total BM cells (n = 6). (D) Frequency of LT‐HSCs (CD48^−^ CD150^+^ LSK) within total BM cells (n = 6). (E) Frequency of MPPs (CD48^+^ CD150^−^ LSK) within total BM cells (n = 6). (F‐H) Mice received Dnak (1 mg, i.p.) or PBS (vehicle). At 24 h post‑injection, LSK cells (Lin^−^ Sca‑1^+^c‑Kit^+^) were FACS‑sorted from bone marrow, and whole‑cell lysates were prepared for Western blotting. (F) O‑GlcNAcylation levels detected by anti‑O‑GlcNAc antibody; GAPDH served as loading control. (G) Protein expression of OGT and OGA. (H) Densitometric quantification of the OGT/OGA ratio (normalized to GAPDH) (n = 3). (I‐K) Mice were treated with PBS, Dnak (1 mg, i.p.), OSMI‑1 plus Dnak (1 mg, i.p.), or Thiamet G plus Dnak (1 mg, i.p.). Bone marrow single‑cell suspensions were prepared 7 days post‑injection and analyzed by flow cytometry (n = 6). (I) Frequency of LSK cells (Lin^−^ c‐Kit^+^ Sca‐1^+^) within total BM cells (n = 6). (J) Frequency of LT‐HSCs (CD48^−^ CD150^+^ LSK) within total BM cells (n = 6). (K) Frequency of MPPs (CD48^+^ CD150^−^ LSK) within total BM cells (n = 6). (L–O) Mice received PBS (vehicle) or Dnak (1 mg, i.p.), and bone marrow extracellular fluids were collected at 24 h post‑injection. Concentrations of IL‑1β (L), TNF‑α (M), G‑CSF (N) and IFN‑γ (O) were measured by ELISA (n = 4). (P–S) Mice were treated with PBS, Dnak (1 mg, i.p.) or IL‐1RA plus Dnak (1 mg, i.p.). Bone marrow single‑cell suspensions were prepared 7 days post‑injection and analyzed by flow cytometry. (P) Representative flow cytometry gating strategy for hematopoietic progenitor cells. After gating on lineage‐negative (Lin^−^) cells, LSK cells were identified as c‐Kit^+^ Sca‐1^+^. Myeloid progenitors (MyPs) were identified as c‐Kit^+^ Sca‐1^−^. (Q) Frequency of LSK cells and MyPs within total BM cells (n = 6). (R) Representative flow cytometry plots for LSK subpopulation analysis. LT‐HSCs were identified as CD48^−^ CD150^+^ within LSK cells; MPPs were identified as CD48^+^ CD150^−^ within LSK cells. (S) Frequency of LT‐HSCs and MPPs within total BM cells (n = 6). Data are presented as mean ± SD. For comparisons between two groups, unpaired two‑tailed Student's t‑test was applied when data met normal distribution assumptions; otherwise, the Mann–Whitney U test was used. Multiple‐group comparisons were analyzed by one‐way ANOVA with Tukey's post hoc test or by Kruskal–Wallis test with Dunn's multiple‐comparison test. ^*^ *p* < 0.05; ^**^ *p* < 0.01; ^***^ *p* < 0.001; ns, not significant.

Consistent with the results of RNA‐seq, MPPs from Dnak‐treated mice displayed increased glucose consumption and lactate production (Figure 3A,B). To determine whether glycolysis is functionally required for Dnak‑induced hematopoietic progenitor expansion, we treated mice with the glycolysis inhibitor 2‑deoxy‑D‑glucose (2‐DG). 2‐DG treatment markedly attenuated the Dnak‑mediated increase in LSK cells, LT‑HSCs, and MPPs (Figure 3C–E). Moreover, LSK cells exhibited elevated levels of O‐GlcNAcylated proteins (Figure 3F–H). To functionally investigate the role of O‑GlcNAcylation in Dnak‑induced myelopoiesis, we employed pharmacological inhibitors of O‐linked N‐acetylglucosamine transferase (OGT) and O‐GlcNAcase (OGA). Treatment with the OGT inhibitor OSMI‑1 selectively suppressed the Dnak‑induced expansion of LSK cells and MPPs, whereas LT‑HSC numbers remained unaffected (Figure 3I–K). Conversely, inhibition of OGA with Thiamet G significantly enhanced the Dnak‑mediated increase in LSK cells, while no notable differences were observed in LT‑HSCs or MPPs (Figure 3I–K). These findings demonstrate that Dnak‐induced trained immunity is mediated by glycolysis and glycosylation, mechanistically identifying O‐GlcNAcylation as a previously unrecognized regulator of central trained immunity.

### 2.5 IL‐1β and TLR4 are Involved in Dnak‐Dependent Training in the BM

To determine whether the effects of Dnak on LSK cells were mediated by cytokines induced in the BM in response to Dnak administration, we performed cytokine analysis in the BM extracellular fluid 24 h following Dnak administration. We found an elevated concentration of IL‐1β, whereas the levels of other cytokines, such as TNF‐α, interferon (IFN)‐γ, and G‐CSF, were not significantly altered (Figure 3L–O). We thus next sought to identify the potential role of IL‐1β in the effects of Dnak on BM progenitor cells. Pharmacologic inhibition of IL‐1 using an IL‐1 receptor antagonist (IL‐1RA; anakinra) prevented the increase of LSK cells and MyPs after Dnak administration (Figure 3P,Q). IL‐1RA also inhibited the Dnak‐induced increase in the frequency of LT‐HSC and MPP cells (Figure 3R,S).

To identify the relationship between O‑GlcNAc modification and IL‐1 signaling, mice were pretreated with OSMI‑1, Thiamet G, or PBS for 2 days, followed by a single intraperitoneal injection of 1 mg Dnak or PBS vehicle. Compared with the Dnak‑alone group, OSMI‑1 pretreatment significantly suppressed Dnak‑induced IL‑1β levels, whereas Thiamet G administration markedly elevated this response (Figure S5A). To investigate whether the TLR4 signaling pathway was involved in the increase of bone marrow IL‐1β levels induced by Dnak, we conducted experiments using TLR4 knockout (TLR4‐/‐) mice. The results showed that 24 h after i.p. injection of Dnak, the bone marrow IL‐1β level in TLR4‐/‐ mice was significantly lower than that in wild‐type (WT) mice (Figure S5B), indicating that Dnak requires TLR4 signaling to elicit the BM cytokine milieu required for HSPC training. In conclusion, we have demonstrated that Dnak can induce trained immunity, and this process requires the involvement of IL‐1β and TLR4.

### 2.6 Pre‐Immunization with Dnak Inhibits Prostate Cancer Growth

**FIGURE 4:**
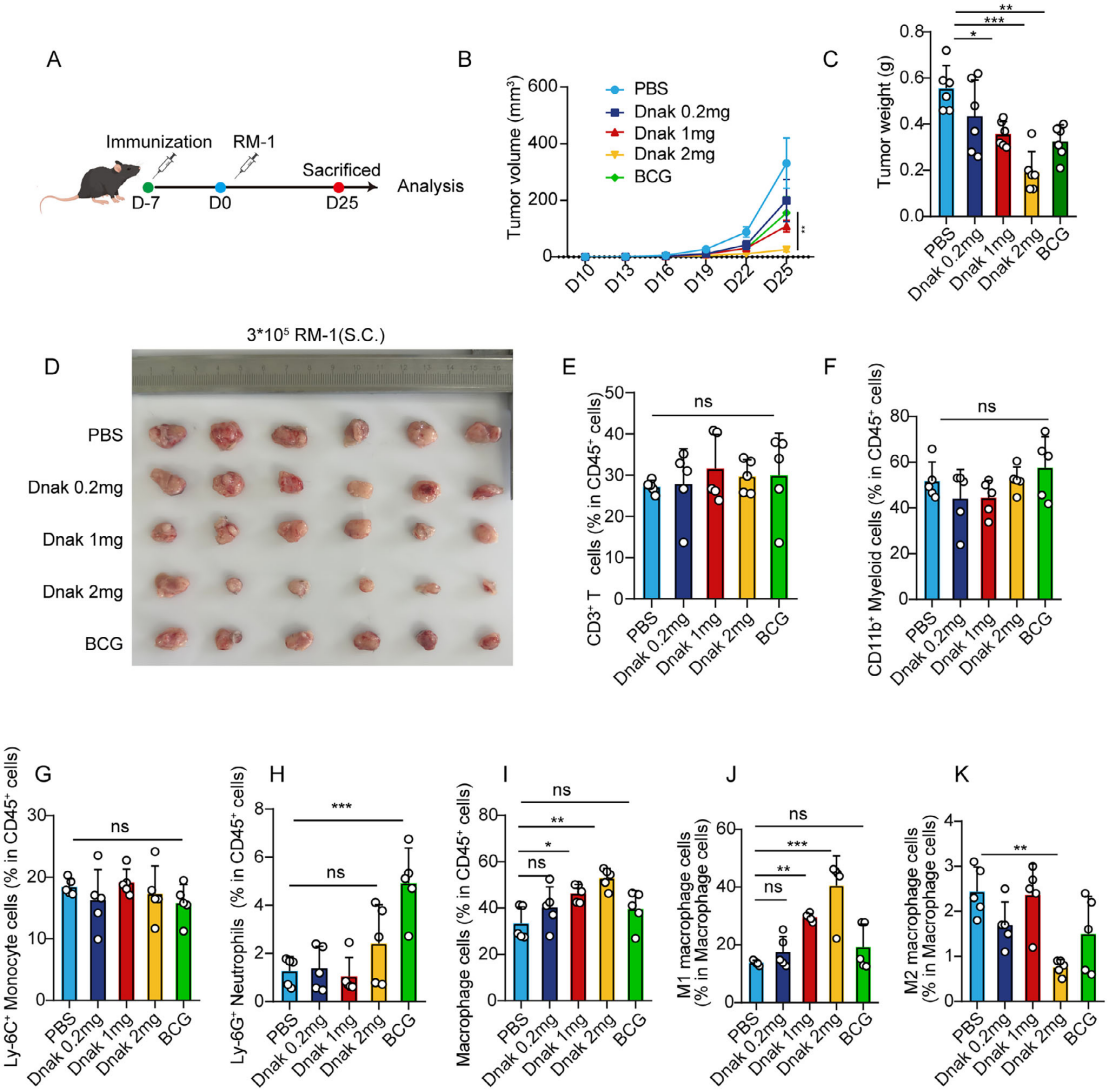
Pre‐immunization with Dnak inhibits prostate cancer growth. (A) WT mice received a single intraperitoneal (i.p.) injection of PBS (vehicle), Dnak at 0.2, 1, or 2 mg per mouse, or an intravenous injection of BCG at 1 × 10^6^ CFU per mouse. Seven days later, all mice were subcutaneously (s.c.) challenged with 3 × 10^5^ RM‑1 cells. Tumor growth was monitored until the experimental endpoint (n = 6). (B) Tumor volume measured over time for mice described in (A) (n = 6). (C) Tumor weight at experimental endpoint for mice described in (A) (n = 6). (D) Representative tumor photographs for mice described in (A) (n = 6). (E–K) Flow‐cytometric analysis of tumor‐infiltrating immune cells in all groups at the experimental endpoint. The detailed gating strategy was shown in Figure S6 (n = 5). (E) Frequency of CD45^+^CD3^+^ T cells within total CD45^+^ leukocytes (n = 5). (F) Frequency of CD45^+^CD11b^+^ myeloid cells within total CD45^+^ leukocytes (n = 5). (G) Frequency of CD45^+^CD11b^+^Ly6G^−^Ly6C^+^ monocytes within total CD45^+^ leukocytes (n = 5). (H) Frequency of CD45^+^CD11b^+^Ly6G^+^Ly6C^−^ neutrophils within total CD45^+^ leukocytes (n = 5). (I) Frequency of CD45^+^CD11b^+^F4/80^+^ macrophages within total CD45^+^ leukocytes (n = 5). (J) Frequency of CD86^+^ CD206^−^ macrophages within CD45^+^CD11b^+^F4/80^+^ macrophages (n = 5). (K) Frequency of CD86^−^ CD206^+^ macrophages within CD45^+^CD11b^+^F4/80^+^ macrophages (n = 5). Data are presented as mean ± SD. For comparisons between two groups, unpaired two‑tailed Student's t‑test was applied when data met normal distribution assumptions; otherwise, the Mann–Whitney U test was used. Multiple‐group comparisons were analyzed by one‐way ANOVA with Tukey's post hoc test or by Kruskal–Wallis test with Dunn's multiple‐comparison test. Tumor growth curves were analyzed using two‐way repeated‐measures ANOVA or mixed‐effects analysis. ^*^ *p* < 0.05; ^**^ *p* < 0.01; ^***^ *p* < 0.001; ns, not significant.

To investigate whether Dnak‑induced trained immunity affects tumor development, WT mice received a single intraperitoneal injection of Dnak (0.2, 1, or 2 mg), with PBS as a vehicle control and BCG (i.v.) as a standard trained‑immunity comparator. Seven days later, all mice were subcutaneously inoculated with RM‐1 prostate cancer cells, and tumor growth was monitored for 25 days (Figure 4A). Dnak treatment reduced tumor growth in a dose‑dependent manner. Mice receiving 1 or 2 mg of Dnak exhibited markedly reduced tumor burdens compared with the 0.2 mg group and PBS‑treated controls, with the highest dose achieving the greatest suppression (Figure 4B–D). As expected, BCG also significantly suppressed tumor growth, confirming its efficacy as a positive control.

To explore the underlying immune mechanisms, we analyzed tumor‑infiltrating leukocytes by flow cytometry. The frequencies of total T cells, myeloid cells, and monocytes were comparable across all groups (Figure 4E–G). In contrast, BCG markedly increased the proportion of intratumoral neutrophils, an effect not observed with any dose of Dnak (Figure 4H). Notably, Dnak treatment dose‑dependently increased the frequency of tumor‑infiltrating macrophages (Figure 4I). Further subtype analysis revealed that Dnak dose‑dependently increased the proportion of M1‑like (CD86^+^ CD206^−^) macrophages and decreased the proportion of M2‑like (CD86^−^ CD206^+^) macrophages, whereas BCG treatment showed no significant difference in either subset compared with PBS controls (Figure 4J,K). To determine whether the observed anti‑tumor effects were model‑independent, we extended our prophylactic regimen to the Tramp C1 prostate cancer model. Consistent with the findings in RM‐1 tumors, Dnak pretreatment inhibited tumor onset and growth, significantly decreasing both tumor volume and weight compared with PBS controls (Figure S7A–C). Flow cytometric profiling of the Tramp C1 tumor microenvironment revealed that Dnak treatment similarly increased the frequency of tumor‑infiltrating macrophages and reduced M2‑like macrophages, closely mirroring the immune alterations observed in the RM‐1 model (Figure S7D–I).

To further assess the therapeutic potential, we used the RM‐1 prostate cancer model, in which mice received Dnak (10, 50, or 100 µg, i.p.), PBS, or BCG (i.v.) after tumor engraftment (Figure S7J). In this therapeutic setting, Dnak dose‑dependently suppressed tumor growth compared to PBS controls (Figure S7K–M).

Collectively, these results indicate that Dnak inhibits prostate cancer growth, potentially through dose‑dependent remodeling of macrophage polarization toward an M1‑skewed phenotype, rather than through neutrophil recruitment, which distinguishes its action from that of BCG‑induced trained immunity.

### 2.7 Trained Immunity Reprograms TAMs to Suppress Prostate Cancer

**FIGURE 5:**
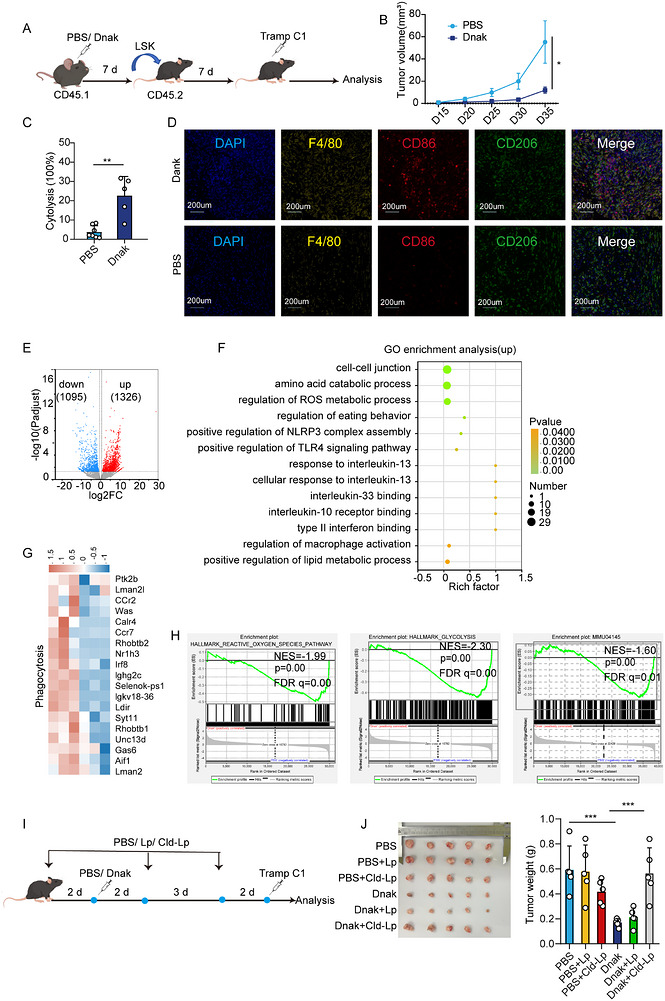
Dnak‐induced trained immunity in bone marrow progenitors reprograms tumor‐associated macrophages to inhibit prostate cancer. (A) Experimental scheme for adoptive transfer. CD45.1 donor mice received a single i.p. injection of Dnak 1 mg or PBS. One week later, LSK cells were sorted from donor BM and transplanted into lethally irradiated CD45.2 recipient mice. Seven days after transplantation, recipients were inoculated s.c. with 2 × 10^6^ Tramp C1 cells. Tumor growth was monitored for 35 days (n = 6). (B) Tumor volume measured over time for mice described in (A) (n = 6). (C) Tumor‐associated macrophages (TAMs) were isolated from mice at the experimental endpoint described in (A). TAMs were co‐cultured with Tramp C1 cells for 48 h. Tumor cell killing was assessed by lactate dehydrogenase (LDH) release assay (n = 7 for PBS group; n = 5 for Dnak group). (D) Representative immunofluorescence staining of tumor sections from mice described in (A). Sections were stained for F4/80 (macrophages, yellow), CD86 (M1‐like, red), CD206 (M2‐like, green), and DAPI (nuclei, blue). Scale bar: 200 µm. (E–H) Transcriptome analysis of TAMs sorted from tumors at the experimental endpoint described in (A) (n = 3 mice per group). (E) Volcano plot showing differential gene expression in TAMs from recipients of Dnak‐trained LSK cells compared to recipients of PBS‐treated LSK cells. Significantly upregulated (red) and downregulated (blue) genes are highlighted (FDR < 0.05). (F) Selected GO biological process terms enriched among upregulated genes. The complete list is provided in Table S5. (G) Heatmap showing expression of genes related to phagocytosis. (H) GSEA plots for reactive oxygen species (ROS) pathway, glycolysis, and phagocytosis gene sets. NES, normalized enrichment score. FDR q‐values are indicated. (I) Experimental scheme for macrophage depletion. WT mice received i.p. injections of clodronate liposomes (Clodrosome) or control liposomes every 4 days, starting 3 days before Dnak or PBS administration. Seven days after Dnak/PBS, mice were inoculated s.c. with Tramp C1 cells. Tumors were harvested at day 35 (n = 5 mice per group). (J) Tumor images (left) and tumor weight (right) at experimental endpoint for mice described in (I) (n = 5). Data are presented as mean ± SD. For comparisons between two groups, an unpaired two‑tailed Student's t‑test was applied when data met normal distribution assumptions; otherwise, the Mann–Whitney U test was used. Multiple‐group comparisons were analyzed by one‐way ANOVA with Tukey's post hoc test or by Kruskal–Wallis test with Dunn's multiple‐comparison test. Tumor growth curves were analyzed using two‐way repeated‐measures ANOVA or mixed‐effects analysis. ^*^ *p* < 0.05; ^**^ *p* < 0.01; ^***^ *p* < 0.001; ns, not significant.

To address whether trained innate immunity mediates anti‐tumor effects on macrophages through modulation of their BM progenitors, we interrogated whether these anti‐tumor effects are transmissible via BM transplantation to non‐trained recipient mice. Donor mice were treated with Dnak or PBS, and 7 days later, their LSK cells were transplanted into non‐trained, lethally irradiated recipient mice. After establishment of hematopoiesis, recipient mice were implanted with Tramp C1 tumors (Figure 5A). Tumor burden was significantly decreased in mice that received LSK cells from Dnak‐trained donors compared to those receiving LSK cells from control‐treated donors (Figure 5B). TAMs from Dnak‐trained mice (hereafter designated “trained” macrophages) or macrophages from control‐treated mice (“non‐trained” macrophages) were co‐cultured with Tramp C1 prostate cancer cells for 48 h, and the release of LDH was detected. Compared to non‐trained macrophages, trained macrophages displayed enhanced tumor cytotoxicity (Figure 5C). To explore the landscape of TAM subsets in Tramp C1 specimens, we performed multiplex immunofluorescence staining. This analysis revealed higher proportions of CD86^+^ macrophages and lower proportions of CD206^+^ macrophages in tumors from Dnak‐treated mice (Figure 5D).

To investigate mechanisms by which macrophages exert anti‐tumor effects upon induction of trained immunity, we conducted RNA sequencing of macrophages sorted from tumor tissues. An altered transcriptomic profile was found in TAMs isolated from the tumors of recipient mice transplanted with BM cells from Dnak‐trained donors, compared to those from control‐treated donors (Figure 5E). GO enrichment analysis demonstrated that pathways involved in macrophage activation were overrepresented (Figure 5F; Table S5). Genes related to phagocytosis in macrophages were upregulated in the “trained” TAM group (Figure 5G). In addition, GSEA revealed a significant positive correlation of “trained” TAMs with signatures of reactive oxygen species (ROS) production, glycolysis, and phagocytosis (Figure 5H; Table S6).

To further determine whether Dnak suppresses Tramp C1 tumor growth through TAMs, we depleted macrophages in vivo using clodronate liposomes (CLP) and evaluated the effect of Dnak on tumor progression. Compared to the control group receiving Dnak alone, macrophage depletion by CLP significantly attenuated the inhibitory effect of Dnak on tumor growth (Figure 5I,J). These results indicate that the antitumor activity of Dnak is, at least in part, dependent on TAMs. Therefore, Dnak induces trained immunity in bone marrow LSK cells, thereby affecting the function of TAMs and inhibiting prostate cancer growth.

### 2.8 Dnak Induces Trained Immunity in BMDMs via Glycolysis

**FIGURE 6:**
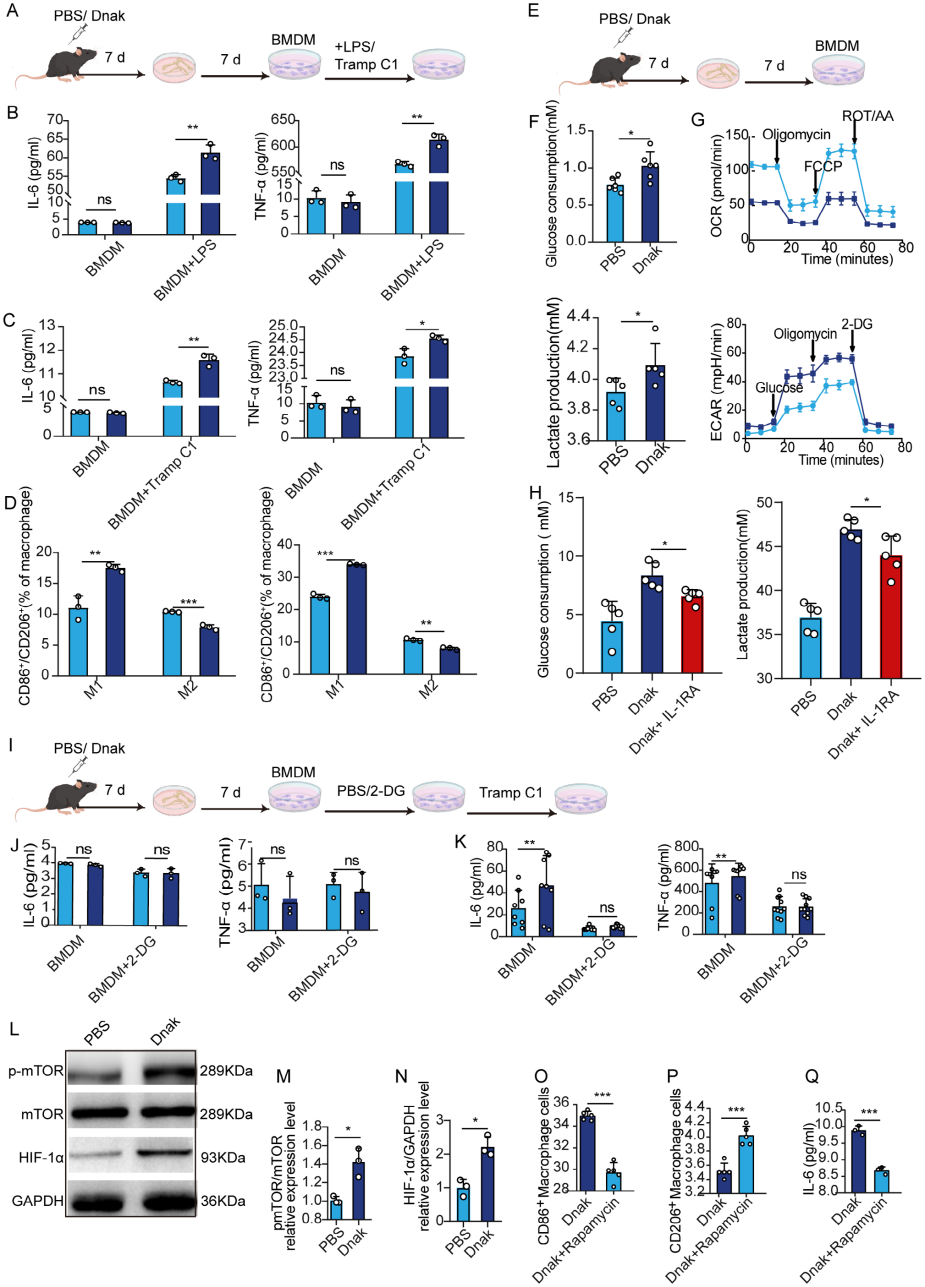
Dnak‐induced trained immunity in bone marrow‐derived macrophages is dependent on glycolysis and the mTOR/HIF‐1α pathway. (A) Experimental scheme for in vitro rechallenge of bone marrow‐derived macrophages (BMDMs). WT mice received a single i.p. injection of Dnak 1 mg or PBS. Seven days later, BM was harvested and differentiated into BMDMs with M‐CSF (20 ng/ml) for 7 days. Differentiated BMDMs were then rechallenged with LPS or Tramp C1 cells for 24 h (n = 3). (B–C) IL‐6 and TNF‐α concentrations in supernatants of BMDMs derived from mice described in (A), before and after rechallenge with LPS (B) or Tramp C1 cells (C) (n = 3). (D) Frequency of M1‐like (CD86^+^) and M2‐like (CD206^+^) cells within BMDMs derived from mice described in (A), before (left)and after (right) rechallenge with Tramp C1 cells (n = 3). (E) Experimental scheme for metabolic analysis. WT mice received Dnak (1 mg, i.p.) or PBS. 7 days later, BM was harvested and differentiated into BMDMs for 7 days. Metabolic assays were performed then. (F) Glucose consumption (up) and lactate production (down) in BMDMs derived from mice described in (E) 24 h after Dnak or PBS administration (n = 6). (G) Oxygen consumption rate (OCR) (up) and extracellular acidification rate (ECAR) (down) measured by Seahorse analysis in BMDMs derived from mice described in (E) (n = 3). (H) WT mice were pretreated with IL‑1RA or PBS for 2 days, followed by intraperitoneal injection of 1 mg Dnak or PBS. Bone marrow cells were collected 7 days after Dnak challenge and differentiated into BMDMs for 7 days. Glucose consumption and lactate production in culture supernatants were measured (n = 6). (I) Experimental scheme for glycolysis inhibition. WT mice received Dnak or PBS. Seven days later, BM was harvested and differentiated into BMDMs with M‐CSF (20 ng/ml) for 7 days. During the final 48 h of differentiation, BMDMs were treated with 2‐DG or vehicle. Cells were then rechallenged with Tramp C1 cells for 24 h (n = 3). (J–K) IL‐6 and TNF‐α concentrations in supernatants of BMDMs derived from mice described in (I), before (J) and after (K) rechallenge with Tramp C1 cells (n = 3). (L–N) WT mice received a single intraperitoneal (i.p.) injection of PBS (vehicle) or Dnak (1 mg). At 7 days post‑injection, bone marrow (BM) cells were harvested and differentiated into BMDMs in the presence of M‑CSF (20 ng/mL) for 7 days. BMDM lysates were then subjected to Western blotting. (L) Representative immunoblots of phospho‑mTOR (p‑mTOR), total mTOR, HIF‑1α, and GAPDH (loading control). (M) Densitometric quantification of the p‑mTOR/total mTOR ratio (normalized to GAPDH; n = 3). (N) Relative protein expression of HIF‑1α (normalized to GAPDH; n = 3). (O–Q) WT mice received Dnak (1 mg, i.p.) or Dnak plus rapamycin. Seven days later, BM was harvested and differentiated into BMDMs. BMDMs were then rechallenged with Tramp C1 cells for 24 h. (O) Frequency of M1‐like (CD86^+^) macrophages within BMDMs after Tramp C1 rechallenge (n = 5). (P) Frequency of M2‐like (CD206^+^) macrophages within BMDMs after Tramp C1 rechallenge (n = 5). (Q) IL‐6 concentrations in supernatants of BMDMs after Tramp C1 rechallenge (n = 5). Data are presented as mean ± SD. For comparisons between two groups, an unpaired two‑tailed Student's t‑test was applied when data met normal distribution assumptions; otherwise, the Mann–Whitney U test was used. Multiple‐group comparisons were analyzed by one‐way ANOVA with Tukey's post hoc test or by Kruskal–Wallis test with Dunn's multiple‐comparison test. ^*^ *p* < 0.05; ^**^ *p* < 0.01; ^***^ *p* < 0.001; ns, not significant.

To determine whether Dnak‐induced changes at the level of LSKs and MPPs could alter the properties of macrophages derived from the BM, we generated bone marrow‐derived macrophages (BMDMs) from the BM of either control (PBS‐i.p.) or Dnak‐i.p. vaccinated WT mice. BMDMs were then challenged with LPS or Tramp C1 cells, and their cytokine secretion was assessed after 24 h (Figure 6A). Macrophages derived from the BM of Dnak‐i.p. vaccinated mice, but not PBS‐i.p. controls, demonstrated significantly higher secretion of IL‐6 and TNF‐α (Figure 6B,C) and a higher M1/M2 ratio (Figure 6D), suggesting the sensitization of BMDMs.

Because Dnak causes MPP cells in mouse bone marrow to exhibit higher glucose consumption and lactate production, and glycolysis is associated with the anti‐tumor phenotype of macrophages [30, 31, 32], we further investigated whether the glucose metabolism of the BMDMs had changed. We found that BMDMs from mice exposed to Dnak showed higher glucose consumption and lactate production, as well as lower oxygen consumption rate (OCR) and higher extracellular acidification rate (ECAR), suggesting a shift from oxidative phosphorylation to glycolysis (Figure 6E–G). Prompted by the established role of IL‐1 in trained immunity (Figure 3P–S), we further investigated its metabolic effects and found that blocking IL‐1 signaling suppressed both glucose consumption and lactate production in bone marrow‐derived macrophages (Figure 6H).

To determine whether the sensitization of BMDMs by Dnak was related to enhanced glycolysis, BMDMs isolated from mice injected with PBS or Dnak were treated with the glycolytic inhibitor 2‐DG prior to challenge with Tramp C1 tumor cells, followed by measurement of cytokine secretion. Following Dnak‐i.p. vaccination, the levels of IL‐6 and TNF‐α secreted by BMDMs stimulated by Tramp C1 cells were significantly higher than those from PBS‐i.p. vaccinated mice, and this enhanced cytokine secretion was inhibited upon 2‐DG treatment (Figure 6I–K).

Since the mTOR/HIF‐1α pathway functions as a metabolic sensor and a key transcription factor regulating macrophage function, we examined its activation in BMDMs [33, 34]. As expected, BMDMs from Dnak‐treated mice exhibited significantly enhanced activation of this pathway compared to those from PBS‐treated controls (Figure 6L–N). Furthermore, treatment with rapamycin, an inhibitor of mTOR, abolished the Dnak‐induced M1 polarization and the enhanced inflammatory response in BMDMs (Figure 6O–Q). Collectively, these results indicate that Dnak potentiates macrophage function through glycolysis, via activation of the mTOR/HIF‐1α pathway, which in turn promotes inflammatory cytokine secretion and M1 polarization.

### 2.9 Dnak Enhances the Efficacy of Prostate Cancer Vaccination

**FIGURE 7:**
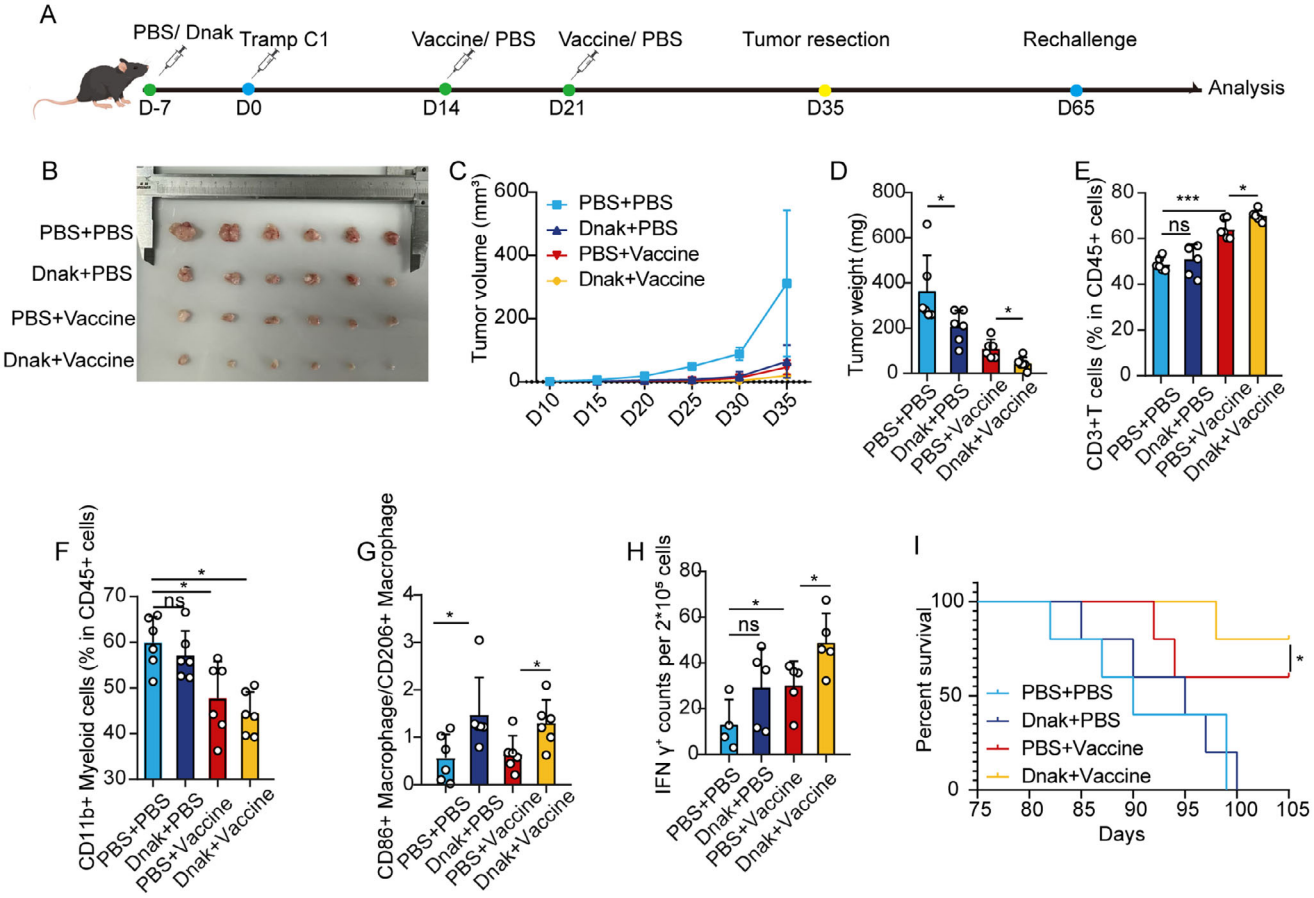
Dnak pretreatment enhances mRNA vaccine‑induced antitumor immunity and establishes long‑term protective memory. (A–I) Mice received a single intraperitoneal injection of PBS or Dnak (1 mg). Seven days later, they were subcutaneously inoculated with 2 × 10^6^ Tramp C1 tumor cells. On days 14 and 21 post‑inoculation, mice were intramuscularly injected with mRNA vaccine (20 µg) or PBS. Tumor growth was monitored until day 35, when tumors were resected for analysis. For long‑term memory evaluation, at day 65, peripheral blood was collected for ELISPOT after TAA restimulation, and mice were rechallenged with Tramp C1 cells to assess tumor‑free survival following rechallenge. (A) Experimental scheme. (B) Resected tumors on day 35 for mice described in (A) (n = 6). (C) Tumor volume at day 35 for mice described in (A) (n = 6). (D) Tumor weight at day 35 for mice described in (A) (n = 6). (E–G) Frequency of CD45^+^CD3^+^ T cells (E), CD45^+^CD11b^+^ myeloid cells (F), and the ratio of CD86^+^ (M1‐like) to CD206^+^ (M2‐like) macrophages within the CD45^+^CD11b^+^F4/80^+^ macrophage population (G) (n = 6). (H) Peripheral blood was collected at day 65, and IFN‑γ ELISpot was performed. Cells were stimulated with TAA (10 µg/mL) for 48 h. Results are presented as spot‑forming cells (SFC) per 10^6^ PBMCs (n = 6). (I) Tumor‐free survival rates in the rechallenge model (n = 5). Data are presented as mean ± SD. For comparisons between two groups, an unpaired two‑tailed Student's t‑test was applied when data met normal distribution assumptions; otherwise, the Mann–Whitney U test was used. Multiple‐group comparisons were analyzed by one‐way ANOVA with Tukey's post hoc test or by Kruskal–Wallis test with Dunn's multiple‐comparison test. Tumor growth curves were analyzed using two‐way repeated‐measures ANOVA or mixed‐effects analysis. Survival curves were compared using the log‐rank test. ^*^ *p* < 0.05; ^**^ *p* < 0.01; ^***^ *p* < 0.001; ns, not significant.

Given the role of trained immunity in reshaping innate immunity, we investigated the combination of Dnak‐induced trained immunity with a tumor vaccine to evaluate the potential synergy between innate and adaptive immunity (Figure 7A). Compared with the tumor vaccine alone, the combination of Dnak and the tumor vaccine significantly suppressed tumor growth (Figure 7B–D). Consistent with our previous findings (Figure 4), Dnak remodeled the tumor microenvironment by reprogramming macrophages, whereas the vaccine promoted T cell infiltration (Figure 7E–G).

Moreover, 30 days after tumor resection, the combination group exhibited stronger T‑cell memory responses in peripheral blood upon antigen restimulation (Figure 7H). In a rechallenge model, the combination therapy also induced more durable immunological memory than the vaccine alone (Figure 7I). Collectively, these findings indicate that trained immunity combined with adaptive immunity achieves superior tumor control.

## 3 Discussion

BCG, as a widely studied inducer of trained immunity, is often thought to exert its effects via MDP‐ mediated activation of the NOD2 receptor [35, 36]. However, the complex composition of the BCG bacillus leaves it unclear whether other components contribute, and its intravenous application poses safety concerns [37, 38, 39]. Therefore, investigating whether other components can induce trained immunity and elucidating their mechanisms is necessary.

This study demonstrates that BCG HSP70 (Dnak) exhibits a superior safety profile compared to other trained immunity inducers and can induce trained immunity. It achieves this by inducing epigenetic, metabolic, and glycosylation changes in bone marrow hematopoietic stem cells (LSKs), ultimately leading to the sensitization of macrophages within the tumor microenvironment and their differentiation toward an M1‐like phenotype, thereby inhibiting prostate cancer growth.

Commonly known inducers of trained immunity include BCG and β‐glucan [40, 41, 42]. To our knowledge, this is the first report identifying HSP70 as a trained immunity inducer that establishes central trained immunity, primarily manifesting as enhanced myelopoiesis in the bone marrow and augmented anti‐infective responses. Dnak‐induced trained immunity is dependent on the TLR4 receptor and bone marrow IL‐1β. This aligns with previous reports highlighting the importance of TLR4 in LPS‐induced trained immunity [28] and IL‐1β in β‐glucan‐induced trained immunity [7, 43], suggesting the potential of HSP70 family members as inducers of trained immunity worthy of further exploration.

The mechanisms of Dnak‐induced trained immunity include epigenetic modifications leading to increased chromatin accessibility and enhanced glycolysis, which are the two most commonly reported mechanisms in trained immunity [44, 45, 46]. Additionally, this study is the first to identify glycosylation as a regulatory mechanism of trained immunity. Glycosylation is a common post‐translational modification involving the covalent attachment of glycans to proteins or other organic molecules [47, 48]. O‐linked β‐D‐N‐acetylglucosamine (O‐GlcNAc) is one of the most common forms, influencing protein folding, stability, activity, and intercellular signaling [49, 50]. This study found that Dnak immunization elevates O‐GlcNAcylation levels in bone marrow MPPs, and that O‐GlcNAcylation is crucial for trained immunity, suggesting that glycosylated proteins might be associated with key proteins or metabolic enzymes involved in inducing trained immunity. Given its emerging role in metabolic sensing and protein function, O‐GlcNAcylation may represent a key node linking nutrient status, epigenetic remodeling, and trained immunity persistence.

The application of trained immunity in anti‐tumor therapy has garnered widespread attention. For instance, Kalafati et al. reported that β‐glucan induces trained immunity in neutrophils, exerting anti‐tumor effects [15], while others recently showed that MDP can improve the tumor microenvironment via trained immunity [16, 51]. Our study reveals that Dnak, by inducing trained immunity in bone marrow LSKs resulting in epigenetic regulation, metabolic changes, and glycosylation—can lead to M1 polarization and sensitization of peripherally differentiated macrophages to inhibit prostate cancer. Given the detrimental impact of malignant tumors on bone marrow cells, particularly myeloid progenitors, leading to the formation of immunosuppressive populations like TAMs and MDSCs in the tumor microenvironment, this induction of trained immunity at the hematopoietic stem cell level presents a novel therapeutic opportunity [52]. In vitro models further confirmed that the observed reprogramming of macrophages following this central trained immunity is dependent on enhanced glycolysis and activation of the mTOR/HIF‐1α pathway, consistent with previous reports by Cheng et al. showing that β‐glucan‐induced peripheral trained immunity in macrophages requires glycolysis [33]. Specifically, mTOR is a well‑established master regulator of glycolytic metabolism, driving the expression of glycolytic enzymes largely through HIF‑1α. This enhanced glycolytic flux increases the production of UDP‑GlcNAc, the essential substrate for O‑GlcNAcylation, thereby directly linking mTOR activation to the O‑GlcNAc modifications we observed.

Nevertheless, this study has some limitations. First, the precise O‐GlcNAcylated targeted proteins contributing to trained immunity were not elucidated and requires further investigation. Second, although Dnak significantly altered the function of tumor microenvironment macrophages, this study did not further explore the impact of these macrophages on other immune cells, particularly T cells. Third, the antitumor efficacy of Dnak was evaluated exclusively in a subcutaneous prostate cancer model; further validation using orthotopic or genetically engineered mouse models will be necessary to confirm its therapeutic potential and clinical relevance.

## 4 Materials and Methods

Detailed methodologies are documented in the Supplementary Materials and Methods.

## Author Contributions

**Kangkang Liu** and **Peng Liu**: conceptualization. **Peng Liu**, **Kangkang Liu**, **Keruo Wang**, **Heyang Guan**, **Taipeng Li**, **Jiaru Hao**, **Zeyuan Wang**, **Yongchao Yan**, **Jiaming Fan**, **Yuan Ma**, and **Yang Yang**: methodology. **Peng Liu** and **Kangkang Liu**: investigation. **Peng Liu**, **Kangkang Liu**, and **Yuanjie Niu**: visualization. **Kangkang Liu**, **Jiatong Chen**, and **Yuanjie Niu**: supervision. **Peng Liu** and **Kangkang Liu**: writing – original draft. **Peng Liu**, **Kangkang Liu**, and **Yuanjie Niu**: writing – review & editing.

## Funding

This study was funded by Chinese Academy of Engineering Science and Technology Cooperation Committee (Grant No. 2024‐DFZD‐03); National Natural Science Foundation of China (Grant No. 82172829; 82403822); Tianjin Health Science and Technology Project (Grant No. TIW12023XK008); Tianjin Haihe Medical Scholar (Grant No. TJSHHYXXZ‐D2‐003).

## Conflicts of Interest

The authors declare no conflicts of interest.

## Supporting information




**Supporting File 1**: advs76886‐sup‐0001‐SuppMat.pdf.

**Supporting File 2**: advs76886‐sup‐0002‐TableS1‐S6.zip.

## Data Availability

The data that support the findings of this study are available on request from the corresponding author. The data are not publicly available due to privacy or ethical restrictions.
