# Supplementary material for: BCG HSP70 Reprograms Macrophages via Central Trained Immunity to Suppress Prostate Cancer
Source: Adv Sci (Weinh). 2026 Aug 5;13(59):e76886. doi: 10.1002/advs.76886 (PMC13440370; doi:10.1002/advs.76886)
Supplement: Supplementary file 1 [file ADVS-13-e76886-s002.pdf]

Supplementary Materials for

• BCG HSP70 Reprograms Macrophages via Central Trained Immunity to Suppress Prostate Cancer

Authors

Peng Liu[1234†], Kangkang Liu[24†], Keruo Wang[234], Heyang Guan[234], Jiaming Fan[234], Taipeng Li[234], Jiaru Hao[234], Zeyuan Wang[234], Yongchao Yan[234], Jiatong Chen[2], Yuan Ma[24], Yang Yang[45], Yuanjie Niu[1234*]

Affiliations

1 Nankai University, Tianjin, 300192, China.

2 Department of Urology, Tianjin Institute of Urology, The Second Hospital of Tianjin Medical University, Tianjin, 300211, China.

3 Tianjin Medical University General Hospital, Tianjin, 300070, China.

4 Haihe Laboratory of Sustainable Chemical Transformations, Tianjin, China.

5 School of Chemical Engineering and Technology, Hebei University of Technology, Tianjin, 300130, China.

* Corresponding author. Yuanjie Niu, Email: niuyuanjie9317@163.com. Address: Nankai University, Tianjin, 300192, China. Tianjin Institute of Urology, The Second Hospital of Tianjin Medical University, Tianjin 300211, China.

† These authors contributed equally to this work.

The PDF file includes:

Materials and Methods
Figure S1 to S11

Other Supplementary Materials for this manuscript include the following:

Tables S1 to S6

Materials and Methods

Recombinant expression of Dnak

The chromosomal DNA of Mycobacterium tuberculosis H37Rv (ATCC: 25618) was used as a template to amplify the ORFs of the Dnak genes. The following primers were designed for amplification:

Gene	Forward primer	Reverse primer
Dnak	CGGCAGCCATATGGCTAGCATGGC TCGTGCGGTCGGG	GGTGGTGGTGGTGCTCGAGTCACT TGGCCTCCCGG

Target DNA bands were excised and digested using restriction enzymes. The resulting fragments (Dnak) were purified and sequenced to confirm their identity. The PET-28a plasmid was linearized by digestion with NheI and XhoI restriction enzymes at 37°C for 2 h. The linearized plasmid was ligated with Dnak gene fragments, with the ligation reaction performed overnight at 16°C. The sequence accuracy of the constructed plasmids PET-28a-Dnak was confirmed by DNA sequencing. The plasmids were transformed into E. coli BL21 (DE-3) competent cells, and protein expression was induced with IPTG. The expressed proteins were purified using a His-tag affinity purification kit, yielding high-purity Dnak (**Figure S8**).

Cells and Cell Culture

Tramp C1 cells were obtained from ATCC and maintained in DMEM supplemented with 10% fetal bovine serum and 1% penicillin-streptomycin. RM-1 cells were obtained from ATCC and maintained in RPMI 1640 medium supplemented with 10% fetal bovine serum and 1% penicillin-streptomycin.

Mice

C57BL/6 CD45.1 mice were obtained from GemPharmatech, and CD45.2 mice were acquired from Sibeifu (Beijing) Experimental Animal Technology Co., Ltd. TLR4 knockout mice (C57BL/6 background, homozygous) were purchased from GemPharmatech (Nanjing, China). All mice were 6-8 weeks old at the start of experiments and maintained under specific-pathogen-free (SPF) conditions in the Laboratory Animal Center of Institute of Radiation Medicine, Chinese Academy of Medical Sciences. Mice were housed in groups of up to five per cage under a 12-hour light/dark cycle, with ambient temperature maintained at 23 ± 2 ° C and relative humidity at 50 ± 10%. All animal experiments were conducted in accordance with institutional guidelines and were approved by the Institutional Animal Care and Use Committee of Institute of Radiation Medicine, Chinese Academy of Medical Sciences. Approval No. (IRM/2-IACUC-2605-011).

Mouse Experiments

Infection Test with Staphylococcus aureus Following LSK Cell Transplantation

C57BL/6 CD45.1 mice received an intraperitoneal injection of 0.2, 1, 2 mg Dnak or PBS (control) in a volume of 100 μL. Bone marrow-derived Lin- Sca-1^{+} c-Kit^{+} (LSK) cells (3,000-5,000 cells per mouse) from CD45.1 donors were transplanted into lethally irradiated CD45.2 recipient mice. Prior to transplantation, LSK cells were co-injected with 3×10^{5} red blood cell-lysed CD45.2 carrier cells. Recipient mice were irradiated with a split dose of 4 Gy followed by a second 4 Gy dose (total 8 Gy). To prevent opportunistic infections, antibiotics were administered in the drinking water starting 24 hours before irradiation and continuing for 1 month thereafter.

For infection challenge following transplantation, Staphylococcus aureus was used. Bacteria were cultured overnight in Luria broth at 37°C, diluted 1:100, and grown to exponential phase ($OD_{600} \approx 3$-4). One week after LSK transplantation, mice were challenged by intraperitoneal injection of 1×10^7 CFU of Staphylococcus aureus in 100 μL sterile PBS. Survival was monitored for 7 days post-infection. For bacterial burden assessment, mice were euthanized 24 hours after infection; spleen, kidney, liver, and lungs were harvested, weighed, and homogenized in PBS. Serial dilutions of homogenates were plated on Staphylococcus aureus Isolation Agar (Difco) containing appropriate antibiotics and incubated overnight at 37° C. Colonies were counted after 16-24 hours.

Biosafety evaluation

Healthy male C57BL/6 mice (6–8 weeks old) were randomly divided into groups and administered PBS (vehicle control), Dnak, MDP, β-glucan, or BCG at three dose levels. For the purified ligands (Dnak, MDP, and β-glucan), the low, intermediate, and high doses were 0.2, 1, and 2 mg per mouse, respectively, all given intraperitoneally (i.p.). For BCG, the corresponding doses were 2×10^5, 1×10^6, and 2×10^6 CFU per mouse, administered intravenously (i.v.). Core body temperature was monitored within the first 24 h post-injection. At 24 h post-injection, tissue samples (liver, kidney, and spleen) were harvested for hematoxylin and eosin (H&E) staining to evaluate organ integrity. Serum biochemical parameters, including alanine aminotransferase (ALT), aspartate aminotransferase (AST), creatinine (Cr), and blood urea nitrogen (BUN), were measured using commercial kits (Nanjing Jiancheng Bioengineering Institute, Nanjing, China) at both 24 h and day 7 post-injection to assess liver and kidney function. For the intermediate-dose group only, serum levels of pro-inflammatory cytokines were further quantified via CBA Multi-factor Detection Technology (Shanghai Shenggong Biotechnology Co., Ltd., Shanghai, China). All procedures were performed according to institutional guidelines.

Samples preparation of bone marrow

To study the effect of Dnak administration on hematopoietic progenitor cells, mice were injected intraperitoneally with 1 mg of Dnak in 100 μL of PBS; control mice received i.p. injections of PBS alone. Mice were euthanized 1 or 7 days later. LSK cells were sorted from mice by FACS for ATAC-seq, and total bone marrow cells were harvested for FACS analysis.

Inhibitor Treatment

IL-1 inhibition was performed by i.p. administration of recombinant IL-1 receptor antagonist (IL-1RA, Anakinra, Amgen GmbH, 100 mg/kg body weight per dose) for one or two days (Cutando et al., 2013). Inhibition of glycolysis was performed by i.p. injection of 2-Deoxy-D-glucose (2-DG) (Sigma; 500 mg/kg body weight per dose) twice on two consecutive days. For OGT inhibition, mice were intraperitoneally (i.p.) administered OSMI-1 at a dose of 0.025 mg per mouse once daily for two consecutive days. For OGA inhibition, mice received Thiamet G (20 mg/kg, i.p.) once daily for two consecutive days. All inhibitor administrations were performed prior to Dnak or PBS treatment.

Tumor Studies

Mice were injected subcutaneously (s.c.) with 2×10^6 Tramp C1 cells or 3×10^5 RM-1 cells into the right flank. To examine the role of trained innate immunity in tumor development, mice were

i.p. treated with a single dose of Dnak (0.2, 1, or 2 mg), with PBS as vehicle control and BCG (1×10^6 CFU) as a standard trained-immunity comparator. Volume of palpable tumors was monitored and calculated using the formula: $(\text{length} \times \text{width}^2) / 2$. Tumor weight and flow cytometry analysis were performed at the end of the experiment.
For adoptive transfer experiments, mice received LSK cell transplantation as described above, and 7 days later, mice were injected s.c. with prostate cancer cells into the right flank. Tumor volume was monitored. Flow sorting and immunofluorescence staining were performed at the end of the experiment.
For macrophage depletion, mice received 200 μL of clodronate liposomes (CLP) or control liposomes (PBS-filled) via i.p. injection every 4 days, starting 3 days before tumor cell inoculation.

Flow Cytometry Analysis

Cell analysis was performed using a FACSCalibur flow cytometer (BD, Heidelberg, Germany). Cell sorting was performed using a FACS Aria II cell sorter (BD, Heidelberg, Germany). Data analysis was performed using FlowJo (Tree Star) software.
For HSC and progenitor analysis, total bone marrow cells were harvested and red blood cells were lysed. The following antibodies were used for staining: anti-Lin-FITC (cocktail including FITC anti-mouse CD3ε, FITC anti-mouse Gr-1, FITC anti-mouse CD11b, FITC anti-mouse CD45R/B220, FITC anti-mouse Ter-119), anti-CD117-APC (clone ACK2), anti-Sca-1-PE (clone D7), anti-CD150-PE/Cyanine7 (clone TC15-12F12.2), anti-CD48-PerCP/Cyanine5.5 (clone HM48-1), anti-CD135-Brilliant Violet 421 (clone A2F10.1), anti-CD16/32-Brilliant Violet 510 (clone 93), anti-CD34-AF700 (clone RAM34). 7-AAD Viability Staining Solution was used as a viability marker. Fluorescence-minus-one (FMO) controls were included for each staining panel to facilitate accurate gate definition, and the gating strategy and FMO plots are provided in **Figure S9**.
To analyze the tumor microenvironment, tumors were homogenized, and single-cell suspensions were prepared after erythrocyte lysis with red blood cell lysis buffer (Solarbio). For cell surface phenotype analysis, anti-CD45 (clone 30-F11), anti-CD3 (clone 17A2), anti-CD11b (clone M1/70), anti-F4/80 (clone QA17A29), anti-Ly6G (clone 1A8), anti-Ly6C (clone HK1.4), anti-CD86 (clone GL-1), and anti-CD206 (clone C068C2) antibodies were used.
Fluorescence-minus-one (FMO) controls were included for each staining panel to facilitate accurate gate definition, and the gating strategy and FMO plots are provided in **Figure S10**.

Vaccine preparation

The prostate cancer mRNA vaccine was designed to encode prostate cancer-associated antigens, including prostate-specific membrane antigen (PSMA) and prostate stem cell antigen (PSCA). Lipid nanoparticle (LNP) raw materials were provided by JenKem Technology Co., Ltd. The synthesis of mRNA and the formulation of the LNP-encapsulated vaccine were performed by CanSino Biologics Inc.

Enzyme-Linked Immunosorbent Assay (ELISA)

Sandwich ELISA was performed to measure cytokine (IL-1β, TNF-α, G-CSF, IFN-γ, IL-6) levels in culture supernatants and BM fluid. Briefly, samples were flash-frozen in liquid nitrogen immediately after harvest and stored at -80°C.

Lactate Dehydrogenase (LDH) Release Assay

Mouse BMDMs were co-cultured with Tramp C1 prostate cancer cells for 24 hours. Tumor cytotoxicity was detected using an LDH Cytotoxicity Assay Kit (Beyotime). Absorbance at 490 nm was measured, and the following groups were established: (1) spontaneous LDH release = LDH value from effector cells alone, (2) low LDH release = LDH value from target cells alone, (3) maximum LDH release = LDH value from target cells lysed with Triton X-100. After subtracting background LDH release (medium alone) from all values, the cytotoxicity was calculated using the following formula: Cytotoxicity (%) = (Experimental LDH release - Spontaneous LDH release - Low LDH release) / (Maximum LDH release - Low LDH release) × 100.

Culture of Bone Marrow-Derived Macrophages (BMDMs)

Bone marrow-derived macrophages (BMDMs) were isolated and cultured as previously described with modifications. Briefly, both hind limbs were collected from mice, and the surrounding muscles were carefully removed. The bones were rinsed with 70% ethanol and transferred into a 60 mm dish containing α-MEM. After cleaning the bones thoroughly, the bone marrow was flushed out slowly with 10 mL of α-MEM using a syringe. The cell suspension was collected into a 50 mL conical tube and centrifuged at 1,260 rpm for 5 minutes at room temperature. The supernatant was discarded, and the pellet was resuspended in α-MEM supplemented with 10% fetal bovine serum (FBS), 100 U/mL penicillin, and 100 μg/mL streptomycin. The cells were then cultured overnight in a humidified incubator at 37°C with 5% CO_2 to allow adherent cells (e.g., fibroblasts and stromal cells) to attach. The next day, non-adherent cells (bone marrow precursors) were collected, counted, and centrifuged. The cell pellet was resuspended at a density of 2×10^7 cells/mL in complete α-MEM (containing 10% FBS, penicillin/streptomycin) supplemented with 20 ng/mL recombinant murine M-CSF. The cell suspension was then distributed into 100 mm culture dishes. Cells were maintained at 37°C in a 5% CO_2 incubator. On day 3 and day 6, the medium was completely removed, the cells were washed once with PBS, and fresh complete α-MEM with M-CSF was added. On day 7, adherent BMDMs were fully differentiated and were harvested by scraping for subsequent experiments.

Western Blot Analysis

Protein expression was assessed using Western blot analysis. Briefly, samples were lysed in RIPA buffer to extract total protein. The lysates were centrifuged, and the supernatants were collected for further analysis. Protein samples were separated by 6-12% SDS-PAGE and transferred onto a PVDF membrane (Millipore). The membrane was blocked with 5% nonfat milk at room temperature for 1 hour, followed by overnight incubation at 4°C with primary antibodies against O-GlcNAc, mTOR, p-mTOR, HIF-1α, OGT, OGA, GAPDH and β-tubulin. After washing, the membrane was incubated with appropriate HRP-conjugated secondary antibodies for 1 hour at room temperature. Protein bands were visualized using enhanced chemiluminescence (ECL) reagent (Thermo Fisher). Western blotting was performed in three independent biological replicates with similar results. The original uncropped blots are provided in **Figure S11**.

H&E Staining

In brief, 4-μm-thick sections were prepared from formalin-fixed paraffin-embedded (FFPE) mouse specimens. After deparaffinization, the sections were stained with hematoxylin for 4 minutes. Nuclei staining was checked under a microscope. Tap water was added to the sections for 45 seconds to allow stain development. Slides were then stained with eosin to visualize the extracellular matrix and cytoplasm for 1 minute. Optical microscopy was employed to visualize and analyze the tissue sections.

Multiplex immunohistochemistry and Multichannel imaging

Immunofluorescence staining was performed on tumor tissue according to the manufacturer's protocol. Tissues were co-stained using the following primary antibodies: F4/80 (70076S, Abmart), CD86 (19589S, Abmart), and CD206 (24595S, Abmart), with DAPI (C1006, Beyotime, China) as a nuclear counterstain. Briefly, tissues were incubated overnight at 4° C with a combination of primary antibodies, followed by incubation with appropriate fluorophore-conjugated secondary antibody mixtures for 1 hour at room temperature. Images were acquired using a fluorescence microscope (Olympus BX51, Tokyo, Japan).

Glucose Consumption and Lactate Production Analysis

The assay was performed after 24 hours of culture. Glucose levels in the media were measured using a Glucose kit (A154-2-1, Nanjing Jiancheng Bioengineering Institute). Glucose consumption was calculated as the glucose concentration in the medium before culture minus that after culture. Lactic acid production was determined using an L-Lactic Acid (L-LA) Content Assay Kit (BC2235, Solarbio). The amount of lactic acid produced was calculated as the lactic acid concentration in the medium after culture minus that before culture.

Seahorse Experiments

Bone marrow-derived macrophages (BMDMs) were isolated from mice that had been treated with either PBS (control) or Dnak for 7 days in vivo and then differentiated ex vivo. For Seahorse analysis, cells were seeded into XFe96 cell culture plates at appropriate densities and allowed to adhere overnight.

On the day prior to the assay, the sensor cartridge was hydrated by adding 200 μL of sterile water to each well of the utility plate and incubated overnight at 37° C. On the day of the assay, the hydration water was replaced with 200 μL of XF Calibrant solution per well, and the cartridge was placed in a non-CO_2 incubator at 37°C for 45-60 minutes. Meanwhile, the assay media were prepared using Agilent Seahorse XF DMEM medium (pH 7.4) supplemented with specific substrates depending on the assay. For the glycolysis stress test, the medium was supplemented with 2 mM glutamine (without glucose). For the mitochondrial stress test, the medium was supplemented with 10 mM glucose, 2 mM glutamine, and 1 mM pyruvate. Cells were washed twice with the respective assay medium, leaving a final volume of 160 μL per well, and then incubated in a non-CO_2 incubator at 37°C for 60 minutes prior to the assay.

The glycolysis stress test was performed using the Seahorse XF Glycolysis Stress Test Kit (103020-100, Agilent). Stock solutions were prepared as follows: glucose (100 mM), oligomycin (100 μM), and 2-deoxy-D-glucose (2-DG, 500 mM). These compounds were diluted in assay medium to achieve 10× working concentrations. The sensor cartridge ports were loaded with 20 μL of glucose (final concentration 10 mM), 22 μL of oligomycin (final concentration 1 μM), and 25 μL of 2-DG (final concentration 50 mM) into ports A, B, and C, respectively.

The mitochondrial stress test was conducted using the Seahorse XF Cell Mito Stress Test Kit (103015-100, Agilent). Stock solutions of oligomycin (100 μM), carbonyl cyanide-p-trifluoromethoxyphenylhydrazone (FCCP, 100 μM), and rotenone/antimycin A (Rot/AA, 50 μM) were prepared. Working solutions were adjusted to 10× final concentrations: oligomycin (15 μM), FCCP (10 μM), and Rot/AA (5 μM). Ports were loaded with 20 μL of oligomycin (final concentration 1.5 μM), 22 μL of FCCP (final concentration 1.0 μM), and 25 μL of Rot/AA (final concentration 0.5 μM) into ports A, B, and C, respectively.

After loading the ports, the sensor cartridge was placed onto the utility plate containing calibrant and calibrated using the Seahorse XFe96 analyzer. Following calibration, the utility plate was replaced with the cell culture plate, and the assay was run according to the standard protocol with repeated cycles of mix, wait, and measure steps. Oxygen consumption rate (OCR) and extracellular acidification rate (ECAR) were automatically calculated by the Seahorse Wave software. At the end of the experiment, cells were lysed for protein quantification, and all data were normalized to total protein content to account for well-to-well variations in cell number.

Statistical analysis

Statistical analyses were performed using SPSS 20.0 and data visualizations created with Origin 6.0. Unless otherwise specified, all experiments included at least three biological replicates with results expressed as mean ± standard deviation (SD). Group comparisons employed either unpaired two-tailed Student's t-tests or one-way ANOVA with Tukey's post hoc testing or by Kruskal–Wallis test with Dunn's multiple-comparison test. Survival curves were analyzed by log-rank test. $^{*}p < 0.05$, $^{**}p < 0.01$, $^{***}p < 0.001$.

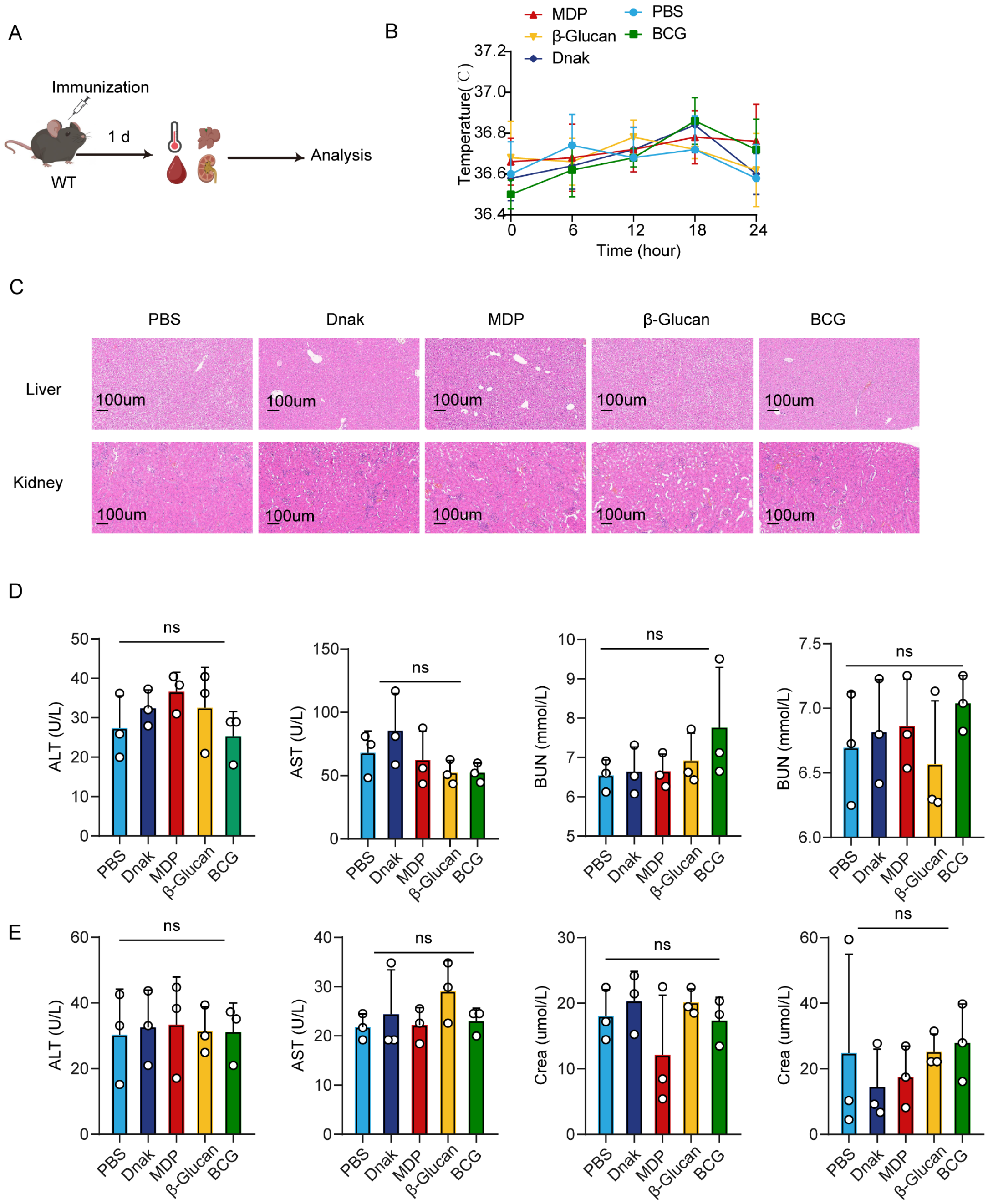

Fig. S1. Biosafety analysis of low-dose Dnak, MDP, β-glucan, and BCG in mice.

(A) Mice were intraperitoneally injected with PBS (vehicle control), Dnak, MDP, or β-glucan (all at 0.2 mg per mouse), or intravenously injected with BCG (2×10^5 CFU per mouse).

(B) Core body temperature was monitored at 24 h post-injection (n=5).

(C) Representative H&E-stained sections of liver and spleen obtained at 24 h post-injection (scale bar = 100 μm).

(D) Serum biomarkers of liver function (ALT, AST) and renal function (CRE, BUN) across treatment groups 24h after treatment (n = 3).

(E) Serum biomarkers of liver function (ALT, AST) and renal function (CRE, BUN) across treatment groups 7 days after treatment (n = 3).

Data are presented as mean ± SD. Multiple-group comparisons were analyzed by one-way ANOVA with Tukey's post hoc test or by Kruskal–Wallis test with Dunn's multiple-comparison test. *$p < 0.05$; **$p < 0.01$; ***$p < 0.001$; ns, not significant.

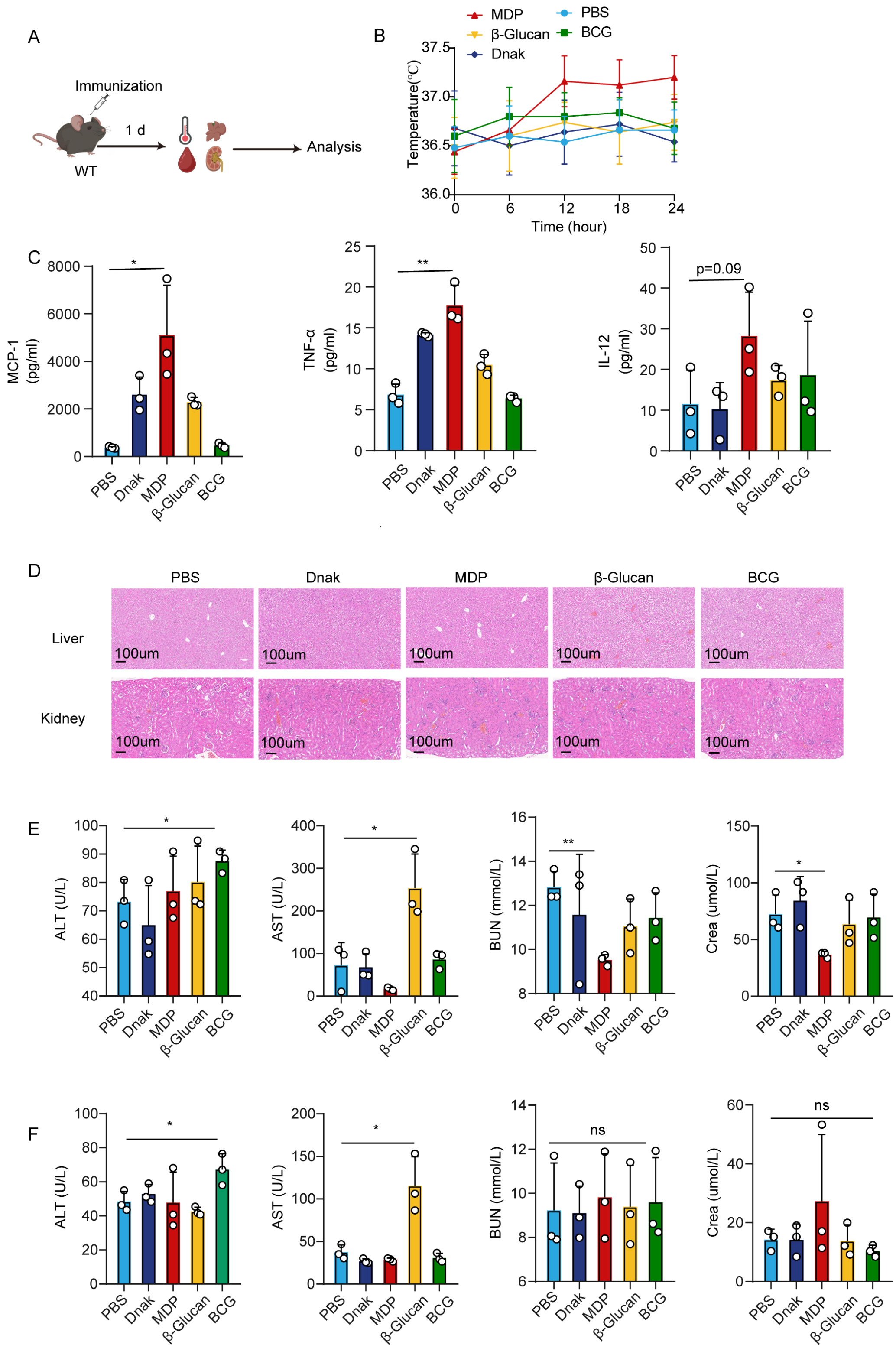

A
Immunization
1 d
WT
Analysis
B
MDP
β-Glucan
Dnak
PBS
BCG
Temperature(℃)
Time (hour)
C
MCP-1 (pg/ml)
TNF-α (pg/ml)
IL-12 (pg/ml)
p=0.09
PBS
Dnak
MDP
β-Glucan
BCG
D
PBS
Dnak
MDP
β-Glucan
BCG
Liver
Kidney
100um
E
ALT (U/L)
AST (U/L)
BUN (mmol/L)
Crea (umol/L)
F
ns

Fig. S2. Biosafety analysis of intermediate -dose Dnak, MDP, β-glucan, and BCG in mice.

(A) Mice were intraperitoneally injected with PBS (vehicle control), Dnak, MDP, or β-glucan (all at 1 mg per mouse), or intravenously injected with BCG (1×10^6 CFU per mouse).
(B) Core body temperature was monitored at 24 h post-injection (n=5).
(C) Serum levels of pro-inflammatory cytokines (e.g., TNF-α, IL-6, and MCP-1) were measured in peripheral blood collected at 24 h post-injection to evaluate cytokine storm (n=3).
(D) Representative H&E-stained sections of liver and spleen obtained at 24 h post-injection (scale bar = 100 μm).
(E) Serum biomarkers of liver function (ALT, AST) and renal function (CRE, BUN) across treatment groups 24h after treatment (n = 3).
(F) Serum biomarkers of liver function (ALT, AST) and renal function (CRE, BUN) across treatment groups 7 days after treatment (n = 3).
Data are presented as mean ± SD. Multiple-group comparisons were analyzed by one-way ANOVA with Tukey's post hoc test or by Kruskal–Wallis test with Dunn's multiple-comparison test. *$p < 0.05$; **$p < 0.01$; ***$p < 0.001$; ns, not significant.

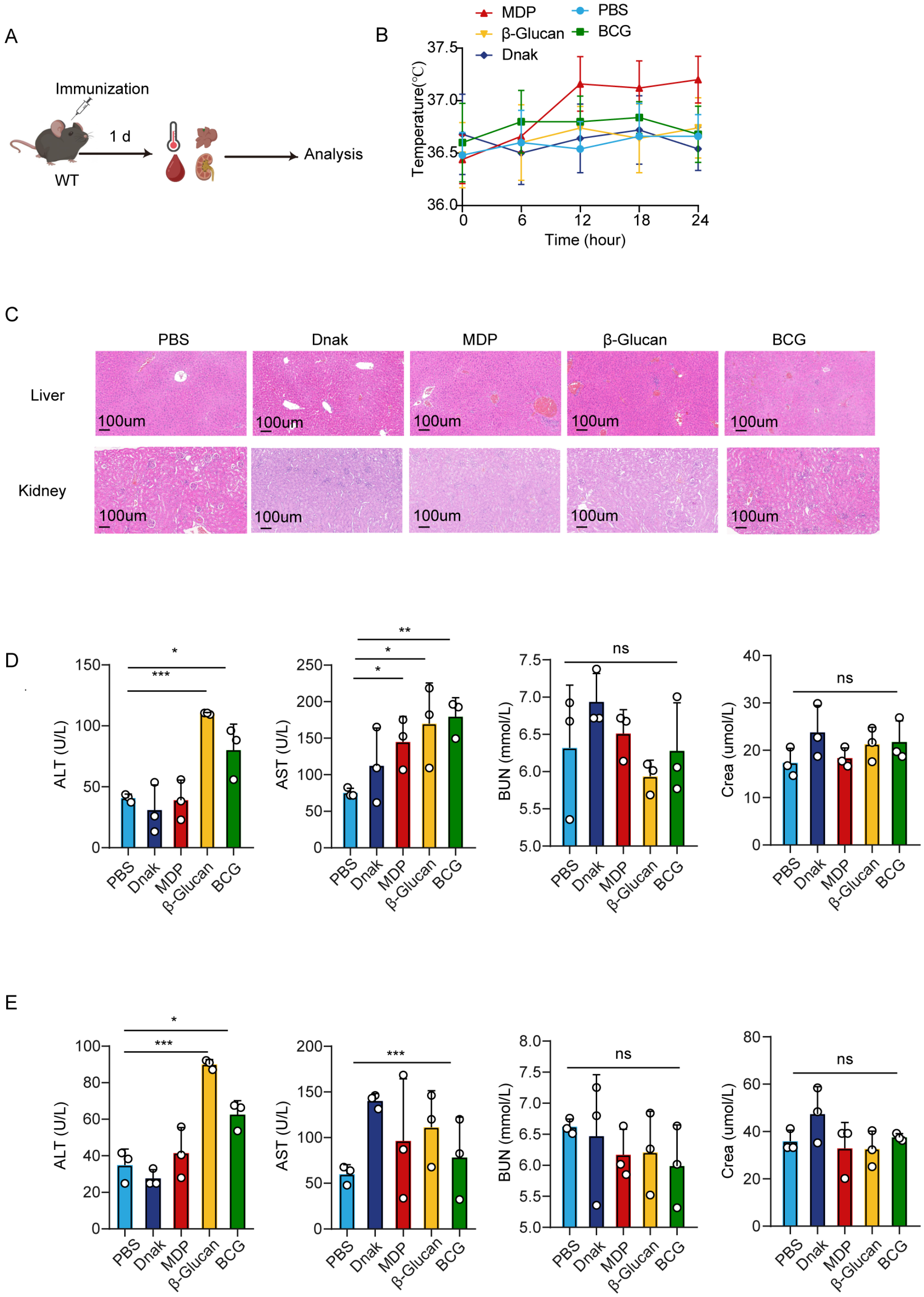

Fig. S3. Biosafety analysis of highest -dose Dnak, MDP, β-glucan, and BCG in mice.

(A) Mice were intraperitoneally injected with PBS (vehicle control), Dnak, MDP, or β-glucan (all at 2 mg per mouse), or intravenously injected with BCG (2×10^6 CFU per mouse).
(B) Core body temperature was monitored at 24 h post-injection (n=5).

(C) Representative H&E-stained sections of liver and spleen obtained at 24 h post-injection (scale bar = 100 μm).
(D) Serum biomarkers of liver function (ALT, AST) and renal function (CRE, BUN) across treatment groups 24h after treatment (n = 3).
(E) Serum biomarkers of liver function (ALT, AST) and renal function (CRE, BUN) across treatment groups 7 days after treatment (n = 3).
Data are presented as mean ± SD. Multiple-group comparisons were analyzed by one-way ANOVA with Tukey's post hoc test or by Kruskal–Wallis test with Dunn's multiple-comparison test. *p < 0.05; **p < 0.01; ***p < 0.001; ns, not significant.

.

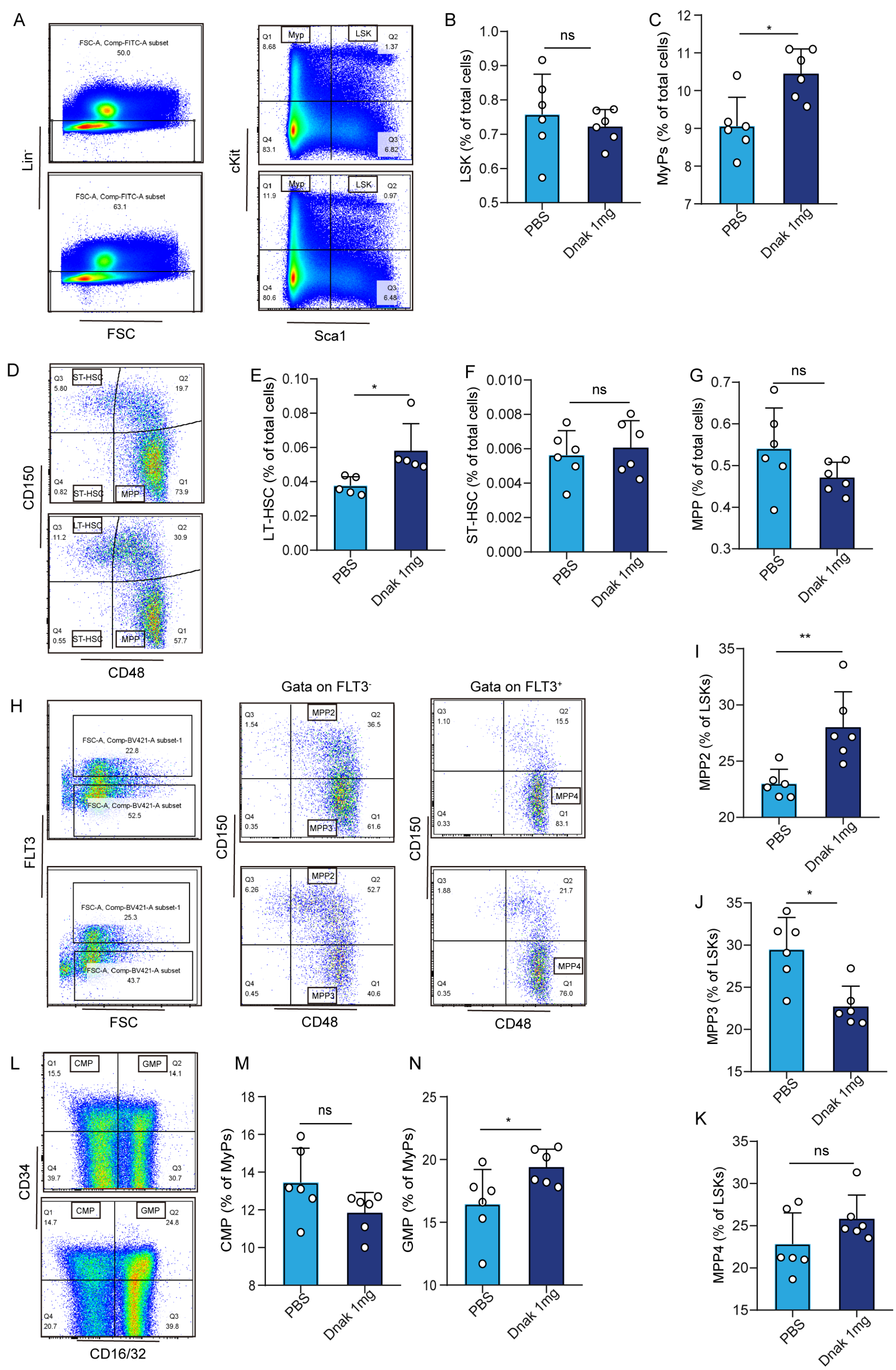
A
Lin⁻
FSC
FSC-A, Comp-FITC-A subset
50.0
FSC-A, Comp-FITC-A subset
63.1
cKit
Sca1
Myp
LSK
Q1 8.68
Q2 1.37
Q3 6.82
Q4 83.1
Q1 11.9
Q2 0.97
Q3 6.48
Q4 80.6
B
LSK (% of total cells)
ns
PBS
Dnak 1mg
C
MyPs (% of total cells)
*
D
CD150
CD48
ST-HSC
MPP
LT-HSC
Q3 5.80
Q2 19.7
Q4 0.82
Q1 73.9
Q3 11.2
Q2 30.9
Q4 0.55
Q1 57.7
E
LT-HSC (% of total cells)
F
ST-HSC (% of total cells)
G
MPP (% of total cells)
H
FLT3
Gata on FLT3⁻
Gata on FLT3⁺
FSC-A, Comp-BV421-A subset-1
22.8
FSC-A, Comp-BV421-A subset
52.5
FSC-A, Comp-BV421-A subset-1
25.3
FSC-A, Comp-BV421-A subset
43.7
MPP2
MPP3
MPP4
I
MPP2 (% of LSKs)
**
J
MPP3 (% of LSKs)
K
MPP4 (% of LSKs)
L
CD34
CD16/32
CMP
GMP
Q1 15.5
Q2 14.1
Q4 39.7
Q3 30.7
Q1 14.7
Q2 24.8
Q4 20.7
Q3 39.8
M
CMP (% of MyPs)
N
GMP (% of MyPs)

Fig. S4. Dnak induces myelopoiesis

(A–K) WT mice were injected with Dnak (1 mg, i.p.) or PBS, and BM analysis was performed after 24 hr.

(A) Representative fluorescence-activated cell sorting (FACS) plots for the identification of hematopoietic progenitor cells. After gating for Lin$^-$ cells, LSK cells were characterized as cKit$^+$ Sca1$^+$ cells. MyP cells were characterized as cKit$^+$ Sca1$^-$ cells.

(B–C) Cell percentages of LSK and MyPs populations in total BM cells of mice at 24 hr after the administration (n = 6).

(D) Representative fluorescence-activated cell sorting (FACS) plots for the identification of hematopoietic progenitor cells. After gating for LSK cells, LT-HSC cells were characterized as CD150$^+$ CD48$^-$ cells. ST-HSC cells were characterized as CD150$^-$ CD48$^-$ cells. MPP cells were characterized as CD150$^-$ CD48$^+$ cells.

(E–G) Frequency of LT-HSC (E), ST-HSC (F) and MPP (G) subpopulations in total cells 24 hr after administration of PBS or Dnak (n = 6).

(H) Representative FACS plots for the identification of MPP subpopulations. After gating for LSK cells, MPP4 cells were characterized as Flt3$^+$CD150$^-$ CD48$^+$LSK, MPP3 cells were characterized as Flt3$^-$ CD150$^-$ CD48$^+$LSK, and MPP2 cells were characterized as Flt3$^-$ CD150$^+$ CD48$^+$LSK.

(I–K) Frequency of MPP2 (I), MPP3 (J) and MPP4 (K) subpopulations in LSK cells 24 hr after administration of PBS or Dnak (n = 6).

(L) Representative FACS plots for the identification of MyP subpopulations. After gating for MyP cells, GMP cells were characterized as CD16/32$^+$ CD34$^+$. CMP cells were characterized as CD16/32$^-$ CD34$^+$.

(M–N) Frequency of CMP (M) and GMP (N) subpopulations in MyPs cells 24 hr after administration of PBS or Dnak (n = 6).

Data are presented as mean ± SD. For comparisons between two groups, unpaired two-tailed Student's t-test was applied when data met normal distribution assumptions; otherwise, the Mann–Whitney U test was used. Multiple-group comparisons were analyzed by one-way ANOVA with Tukey's post hoc test or by Kruskal–Wallis test with Dunn's multiple-comparison test. $*p < 0.05$; $**p < 0.01$; $***p < 0.001$; ns, not significant.

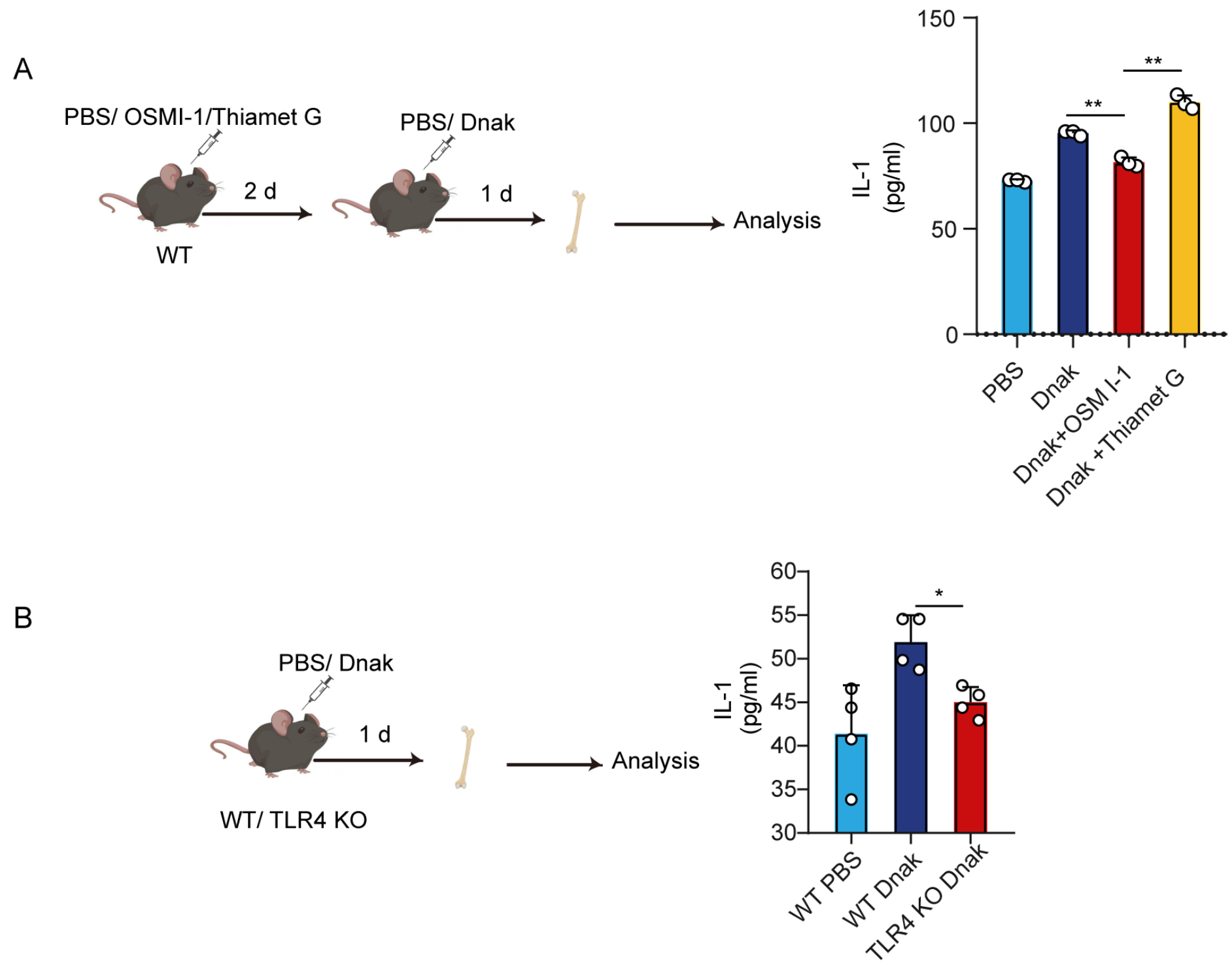

Fig. S5. O-GlcNAcylation and TLR4 are required for Dnak-induced IL-1β production in bone marrow

(A) Mice were pretreated with OSMI-1 (O-GlcNAc transferase inhibitor), Thiamet G (O-GlcNAcase inhibitor), or PBS for 2 days, then received a single intraperitoneal injection of 1 mg Dnak or PBS vehicle. Bone marrow extracellular fluid was collected 24 h later, and IL-1β concentrations were measured by ELISA ($n = 3$).

(B) IL-1β concentration in BM extracellular fluid 24 hours after PBS or Dnak administration in wild-type (WT) and TLR4 knockout (TLR4−/−) mice ($n = 4$).

Data are presented as mean ± SD. For comparisons between two groups, unpaired two-tailed Student's t-test was applied when data met normal distribution assumptions; otherwise, the Mann–Whitney U test was used. Multiple-group comparisons were analyzed by one-way ANOVA with Tukey's post hoc test or by Kruskal–Wallis test with Dunn's multiple-comparison test. $*p < 0.05$; $**p < 0.01$; $***p < 0.001$; ns, not significant.

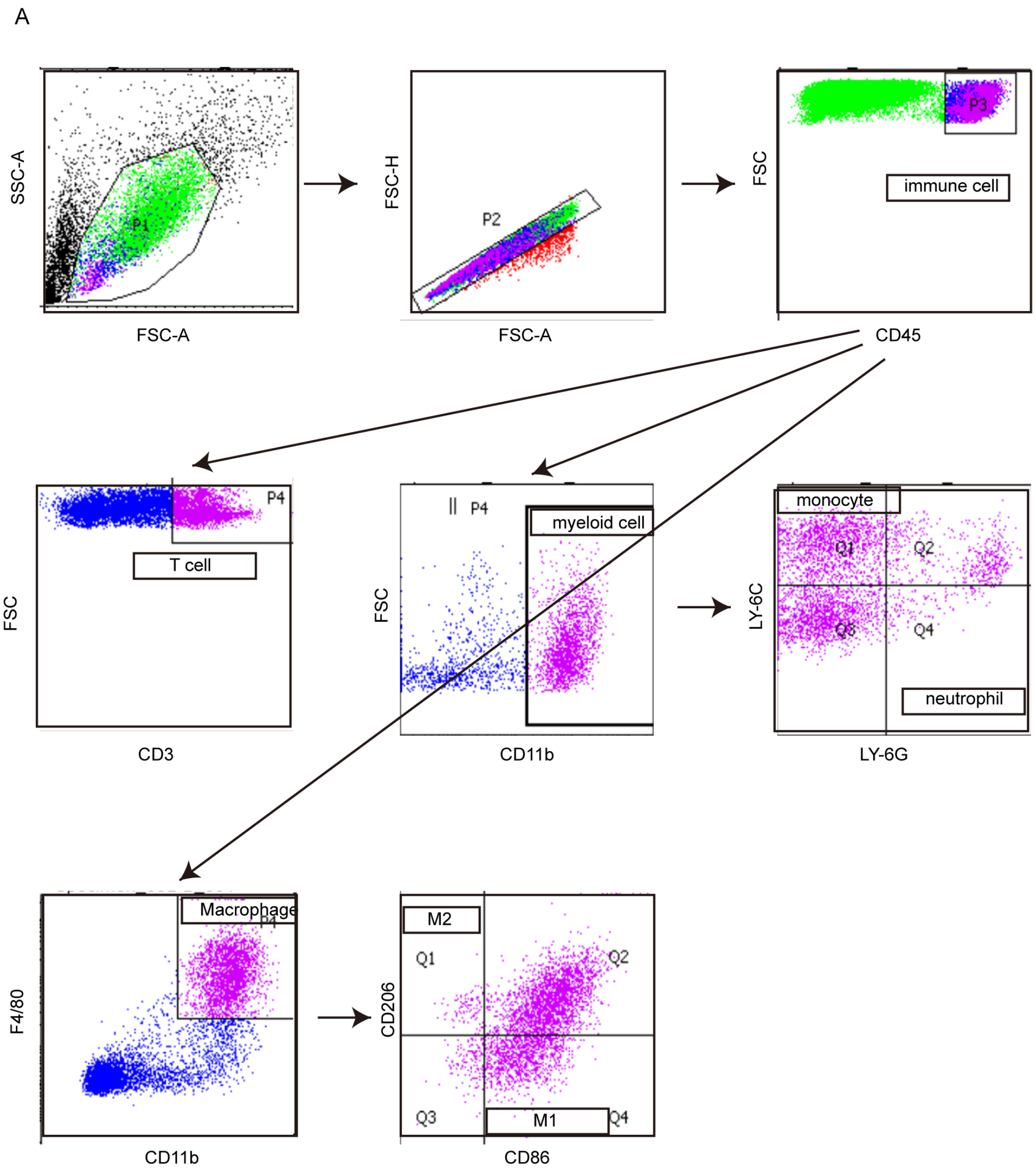

Fig. S6. Gating strategy for flow cytometric analysis of tumor-infiltrating immune cells.

(A) Representative flow cytometric gating strategy for tumor-infiltrating immune cells. Tumor tissues were harvested at the experimental endpoint, dissociated into single-cell suspensions, and stained with fluorochrome-conjugated antibodies. Immune cells were first gated as $CD45^+$ leukocytes. Among the $CD45^+$ population, T cells were defined as $CD45^+CD3^+$, and myeloid cells were defined as $CD45^+CD11b^+$. Within the myeloid compartment, neutrophils were identified as $CD45^+CD11b^+Ly6G^+Ly6C^-$, monocytes as $CD45^+CD11b^+Ly6G^-Ly6C^+$, and macrophages as $CD45^+CD11b^+F4/80^+$. Tumor-associated macrophages (TAMs) were further characterized by their polarization status: M1-like macrophages were defined as

$CD45^+CD11b^+F4/80^+CD86^+CD206^-$, and M2-like macrophages as $CD45^+CD11b^+F4/80^+CD86^-CD206^+$.

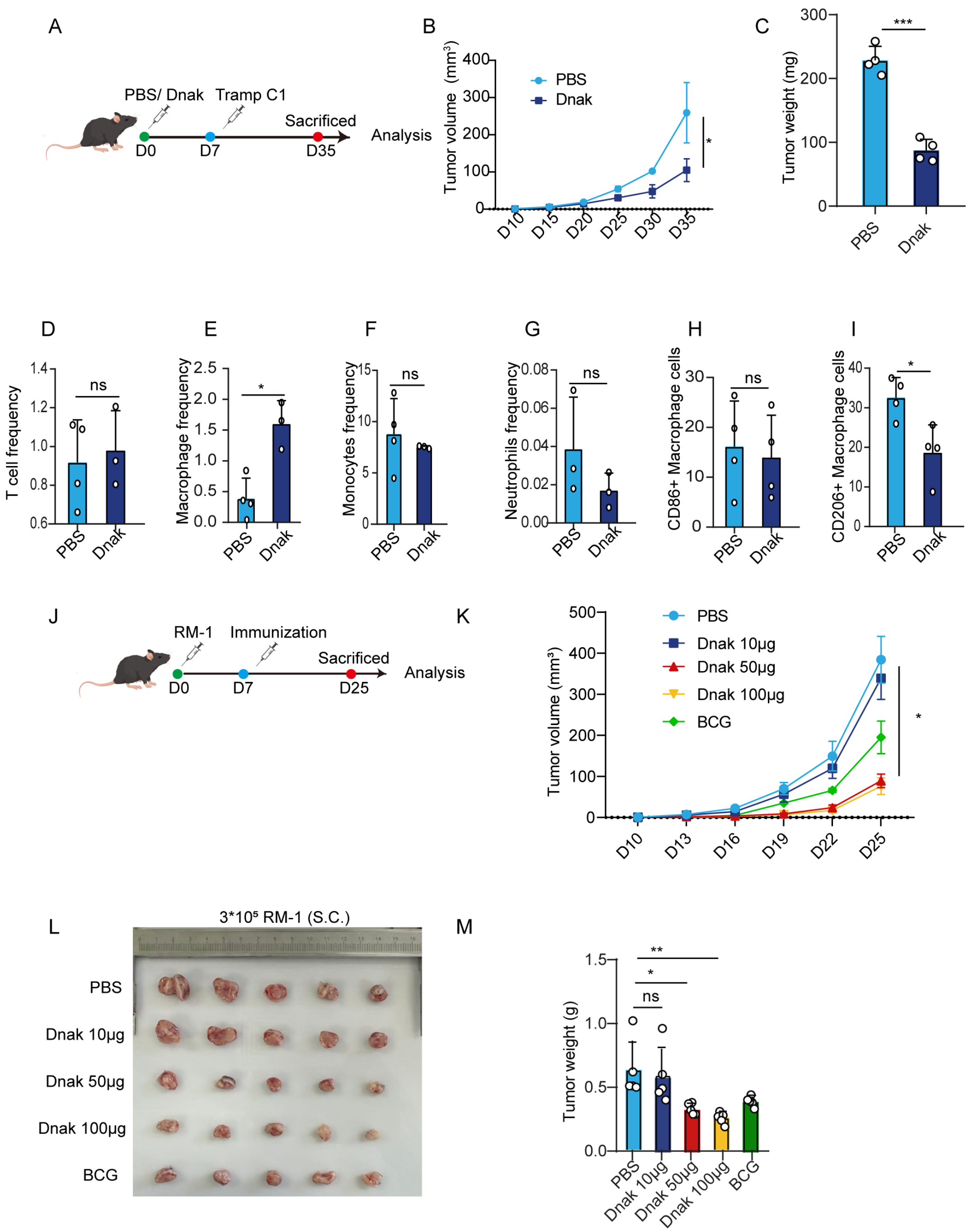

Fig. S7. Dnak inhibits prostate cancer growth.

(A-I) WT mice received a single intraperitoneal (i.p.) injection of PBS (vehicle) or Dnak (1 mg per mouse). Seven days later, all mice were subcutaneously (s.c.) challenged with 2×10^6 Tramp C1 cells. Tumor growth was monitored until the experimental endpoint, at which time tumor tissues were harvested for flow cytometric analysis of the tumor microenvironment (n = 4).

(A) Experimental scheme.
(B) Tumor volume measured over time (n = 4).
(C) Tumor weight at experimental endpoint (day 35) (n = 4).
(D–I) Flow-cytometric analysis of tumor-infiltrating immune cells at the experimental endpoint. The detailed gating strategy was shown in **Figure S6**. Frequency of $CD45^+CD3^+$ T cells (D), $CD45^+CD11b^+F4/80^+$ macrophages (E), $CD45^+CD11b^+Ly6G^-Ly6C^+$ monocytes (F), and $CD45^+CD11b^+Ly6G^+Ly6C^-$ neutrophils (G) within total $CD45^+$ leukocytes. Frequencies were normalized to spleen controls (n = 4).
(H–I) Frequency of $CD86^+$ (M1-like) macrophages (H) and $CD206^+$ (M2-like) macrophages (I) within the $CD45^+CD11b^+F4/80^+$ macrophage population (n = 4).
(J-M) WT mice were subcutaneously (s.c.) inoculated with 3×10^5 RM-1 cells and then received Subcutaneous injections of PBS (vehicle), Dnak at 10, 50, or 100 μg per mouse, or intravenous (i.v.) injection of BCG at 1×10^6 CFU per mouse, as described in (J). Tumor growth was monitored until the endpoint, and tumor volumes were recorded over time (K). At the endpoint, tumors were excised, photographed, and weighed (L, M) (n = 5).
Data are presented as mean ± SD. For comparisons between two groups, unpaired two-tailed Student's t-test was applied when data met normal distribution assumptions; otherwise, the Mann–Whitney U test was used. Multiple-group comparisons were analyzed by one-way ANOVA with Tukey's post hoc test or by Kruskal–Wallis test with Dunn's multiple-comparison test. Tumor growth curves were analyzed using two-way repeated-measures ANOVA or mixed-effects analysis. *$p < 0.05$; **$p < 0.01$; ***$p < 0.001$; ns, not significant.

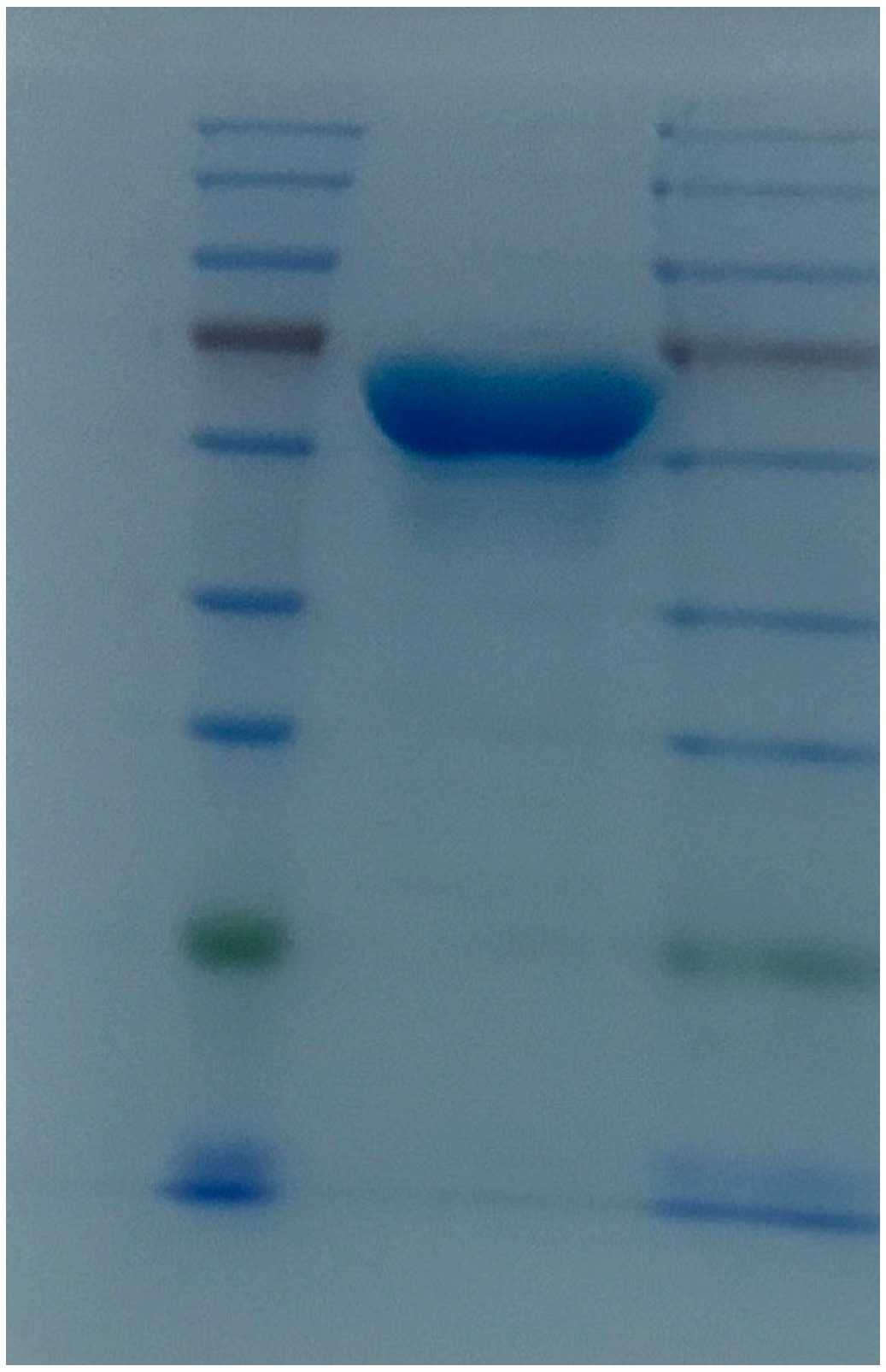

Fig. S8. Expression and purification of recombinant Dnak

(A) SDS-PAGE analysis of Dnak.

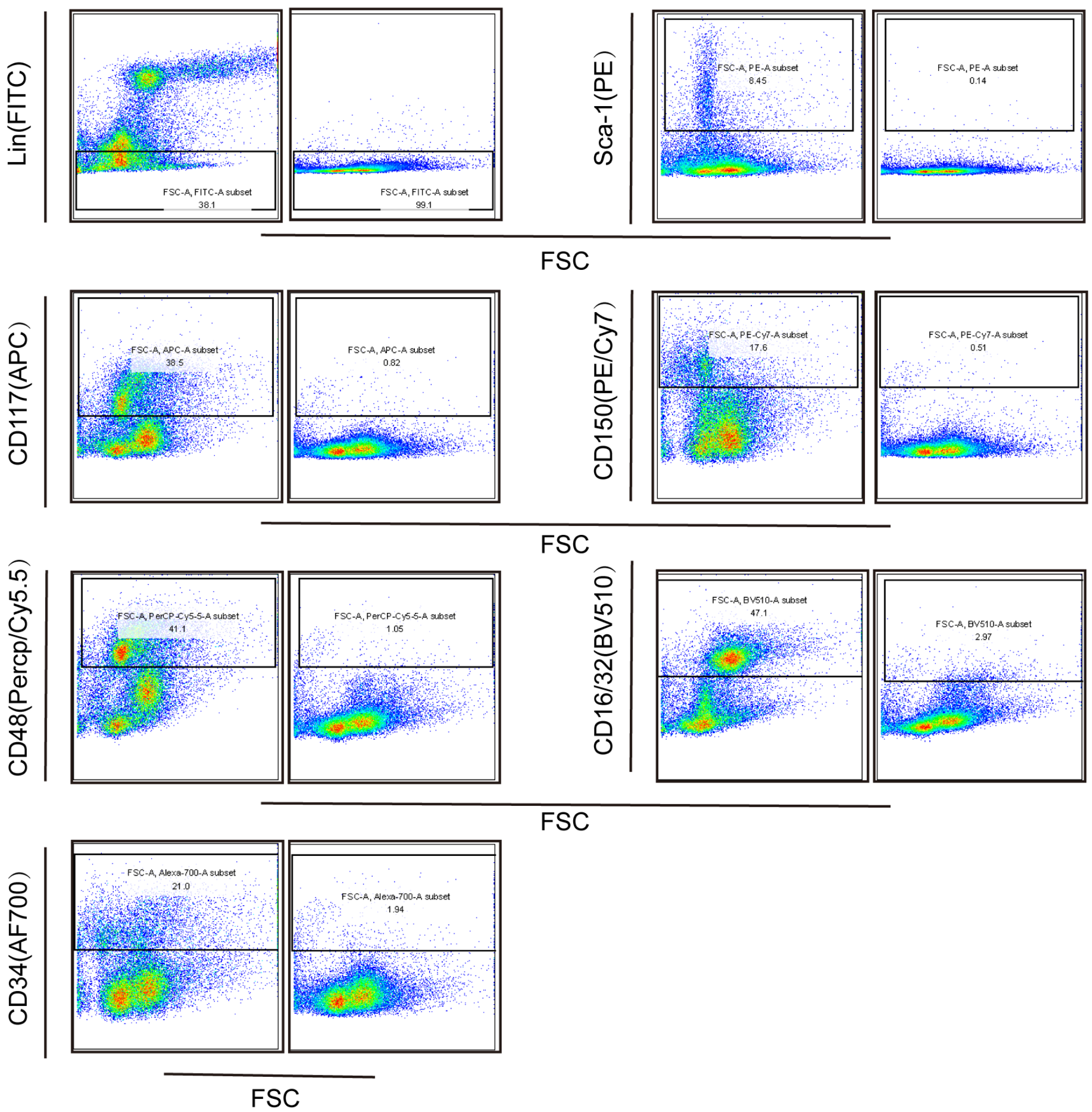

Fig. S9. FMO controls for bone marrow HSC and progenitor analysis.

(A) Fluorescence-minus-one (FMO) controls for each fluorochrome-conjugated antibody used in the panel, demonstrating the specificity of staining and defining the boundaries for positive/negative populations.

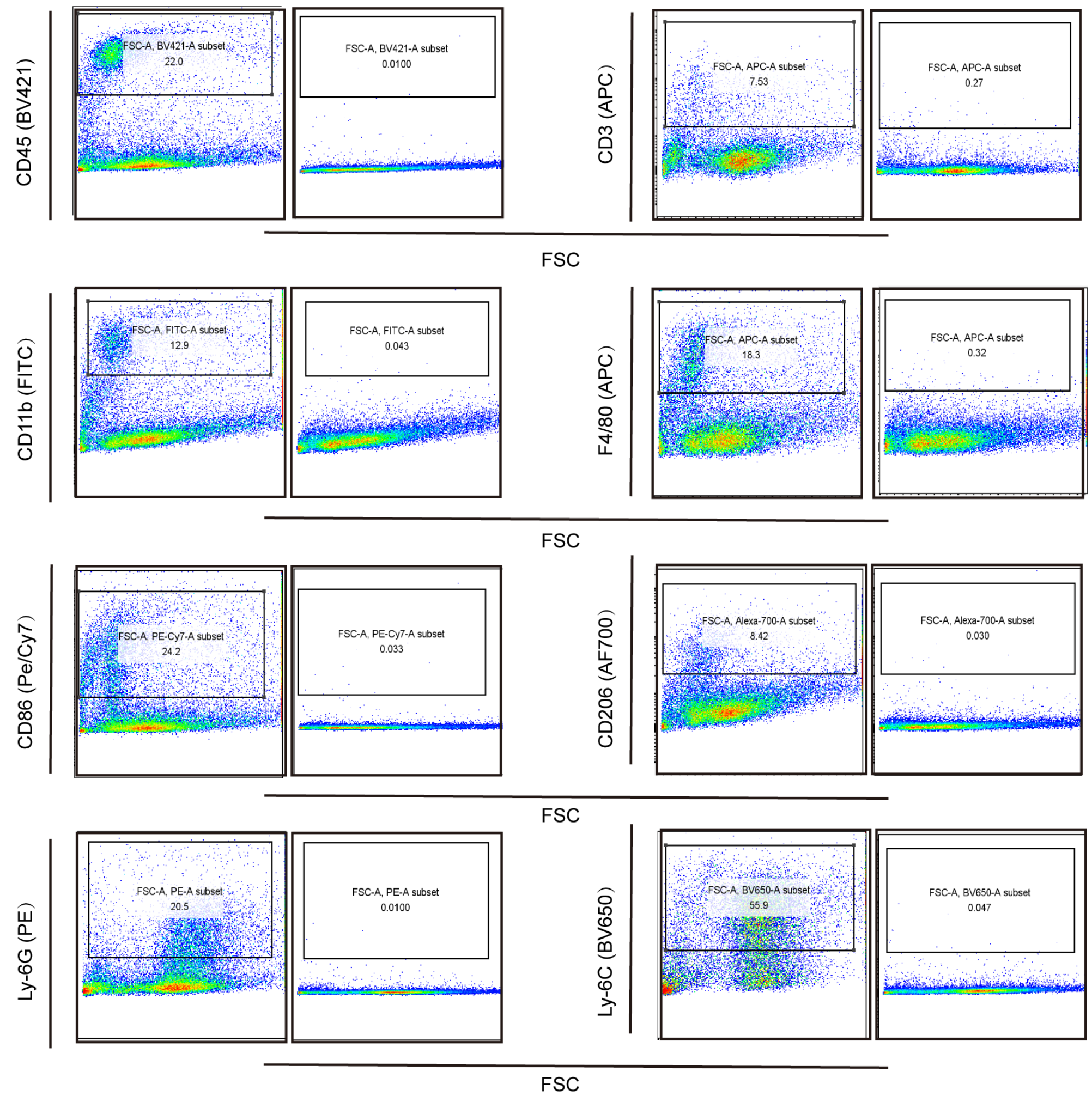

Fig. S10. FMO controls for immune cells in the tumor microenvironment.

(A) Fluorescence-minus-one (FMO) controls for each fluorochrome-conjugated antibody used in the panel, demonstrating the specificity of staining and defining the boundaries for positive/negative populations.

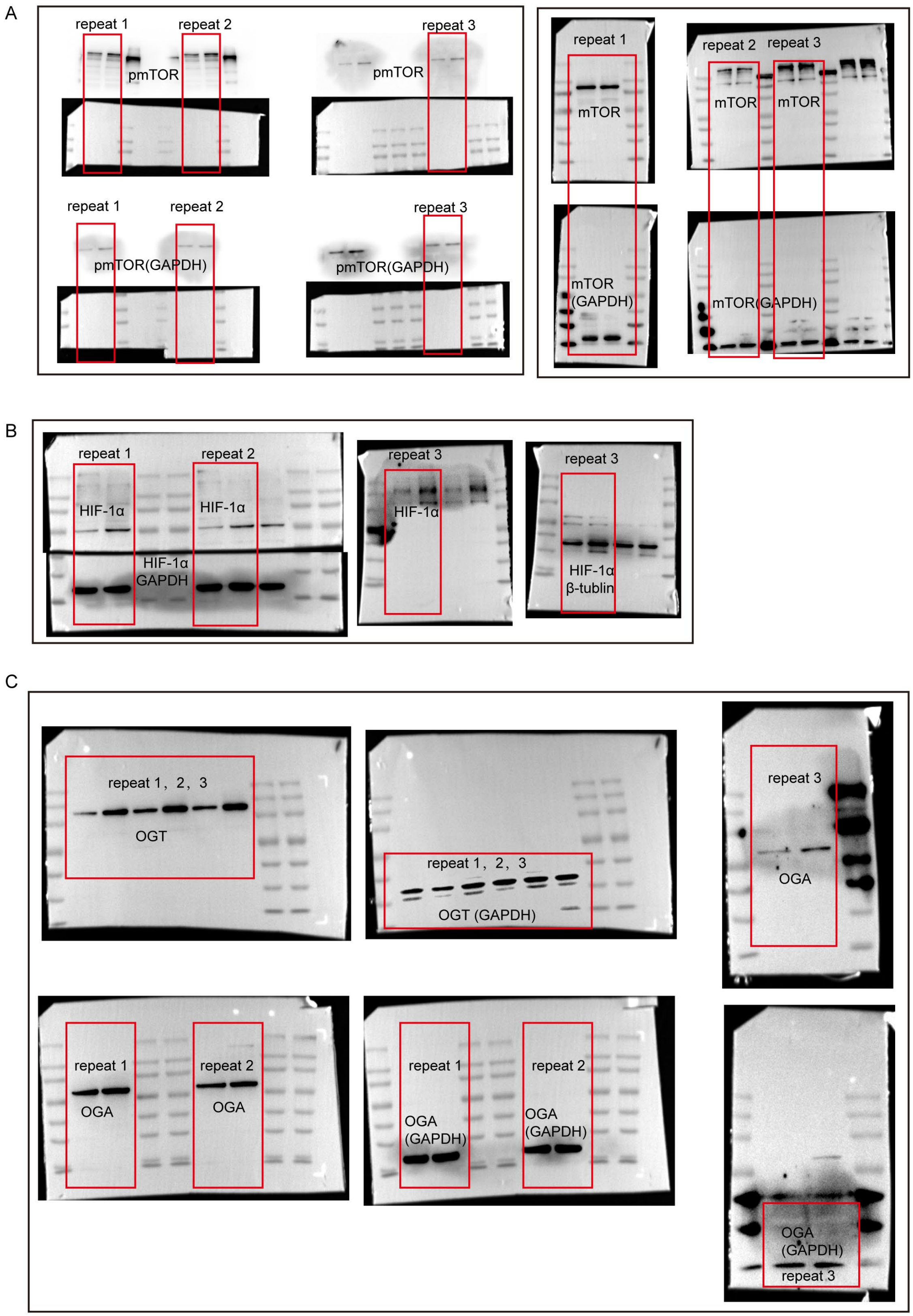
A
repeat 1
repeat 2
pmTOR
repeat 3
pmTOR
repeat 1
repeat 2
pmTOR(GAPDH)
repeat 3
pmTOR(GAPDH)
repeat 1
mTOR
mTOR
(GAPDH)
repeat 2
repeat 3
mTOR
mTOR
mTOR(GAPDH)
B
repeat 1
repeat 2
HIF-1α
HIF-1α
HIF-1α
GAPDH
repeat 3
HIF-1α
repeat 3
HIF-1α
β-tublin
C
repeat 1，2，3
OGT
repeat 1，2，3
OGT (GAPDH)
repeat 3
OGA
repeat 1
OGA
repeat 2
OGA
repeat 1
OGA
(GAPDH)
repeat 2
OGA
(GAPDH)
OGA
(GAPDH)
repeat 3

Fig. S11. Uncropped Western blot membranes

(A) Full-length scans of the original Western blot membranes for (A) p-mTOR and mTOR, (B) HIF-1α, and (C) OGT and OGA. Each blot is from three independent biological replicates (n = 3), and the cropped regions presented in the main figure are indicated by black boxes. Membranes were sequentially probed with the indicated antibodies; GAPDH or β-tubulin served as a loading control.

Table S1. (separate file)
Gene ontology enrichment of different peaks associated genes.

Table S2. (separate file)
Homer Known Motif Enrichment Results

Table S3. (separate file)
Gene ontology enrichment results of MPP RNA-seq

Table S4. (separate file)
Gene set enrichment analysis of MPP RNA-seq

Table S5. (separate file)
Gene ontology enrichment results of macrophage RNA-seq

Table S6. (separate file)
Gene set enrichment analysis of macrophage RNA-seq
